# Physiological roles of phosphoinositides and inositol phosphates: Implications for metabolic dysfunction-associated steatotic liver disease

**DOI:** 10.1042/CS20257631

**Published:** 2025-10-01

**Authors:** Zhili Cheng, Magdalene K Montgomery

**Affiliations:** 1 Department of Anatomy and Physiology, School of Biomedical Sciences, Faculty of Medicine Dentistry and Health Sciences, The University of Melbourne, Melbourne, VIC, 3010, Australia

**Keywords:** inositol polyphosphates, inositol trisphosphate, inositol trisphosphate receptor, lipid metabolism, metabolism, non-alcoholic fatty liver disease, phosphatidylinositol

## Abstract

Phosphoinositides and inositol phosphates (IPs) are integral to numerous cellular processes, including membrane trafficking, signal transduction and calcium dynamics. These lipid-derived signalling mediators orchestrate the spatial and temporal regulation of many signalling cascades, largely through interactions with specific effector proteins. Recent studies have highlighted their critical roles in metabolic homeostasis and the pathogenesis of metabolic dysfunction-associated steatotic liver disease (MASLD). In this review, we examine the pathways important for phosphoinositide and IP synthesis, and the physiological functions of myo-inositol, d-chiro-inositol and phosphatidylinositol, as well as their phosphorylated inositol counterparts, including phosphoinositides (PI(3)P, PI(4)P, PI(3,4)P2, PI(3,5)P2, PI(4,5)P2, PI(3,4,5)P3) and IPs (inositol 1,4,5-trisphosphate (IP3), inositol 1,3,4,5-tetrakisphosphate (IP4), inositol pentakisphosphate (IP5), inositol hexaphosphate (IP6 or phytic acid) and inositol pyrophosphates (IP7 and IP8)), with an emphasis on their emerging significance in hepatic metabolism. We explore how perturbations in IP metabolism contribute to the development and progression of MASLD, liver inflammation, fibrosis and hepatic insulin resistance. We further highlight recent studies utilizing genetic models and pharmacological interventions that underscore the therapeutic potential of targeting inositol metabolism in MASLD. This review synthesizes current knowledge to provide a comprehensive understanding of how phosphoinositides and IPs integrate metabolic cues and contribute to hepatic pathophysiology, identifying knowledge gaps and offering novel insights for therapeutic innovation in the management of MASLD.

## Introduction

### Introduction into inositol phosphate and phosphoinositide metabolism

Inositol phosphates (IPs) and phosphoinositides comprise a diverse family of intracellular signalling molecules derived from myo-inositol (MI, historically termed vitamin B_8_), a six-carbon cyclic polyol (sugar alcohol) that can be linked to a lipid backbone to generate phosphatidylinositol (PI), which is subsequently phosphorylated at several positions within the inositol ring to generate a range of phosphoinositides, with the best characterized being phosphatidylinositol (3, 4, 5) -trisphosphate (PI (3, 4, 5 P3) or PIP3). The hydrolysis of phosphoinositides or the incremental phosphorylation on the six hydroxyl groups of the inositol ring leads to the formation of mono- to poly-phosphorylated inositol derivatives, including IP3 (Inositol 1,4,5-trisphosphate), IP4, IP5 and IP6 (Phytic acid), as well as inositol pyrophosphates that contain high-energy pyrophosphate bonds (e.g. IP7, IP8) [1]. MI is not an essential nutrient for humans, as it can be derived from glucose-6-phosphate via the enzyme inositol-3-phosphate synthase or through dephosphorylation reactions by specific inositol phosphatases forming free MI [2]. While MI can also be obtained from inositol-rich foods like grains and beans [3], the widespread consumption of refined foods has decreased the dietary availability of MI and IPs, while simultaneously increasing metabolic demands for these molecules due to higher intakes of sugar, refined carbohydrates and caffeine [4–8]. This imbalance between reduced intake and increased demand underscores the need to better understand the metabolic fate of MI and its derivatives.

The stereochemical configuration of inositol is stabilized during biosynthesis, resulting in the predominant MI isoform, which is the most stable and abundant in biological systems [9]. A secondary physiologically relevant stereo-isoform is D-chiro-inositol (DCI), which is derived from MI via an insulin-dependent epimerization reaction [10]. The distribution of these isomers across organs is tissue-specific and reflects differing metabolic needs. MI is enriched in tissues with high glucose utilization, such as the brain, heart and ovaries, while D-chiro-inositol predominates in tissues associated with glucose storage and glycogen metabolism, including the liver and skeletal muscle [6].

Historically, IPs were regarded primarily as intermediates in the recycling of MI, serving as storage or metabolic reservoirs. However, emerging evidence over the last two decades has highlighted the physiological relevance of MI, D-chiro-inositol and IPs in cellular signalling and metabolic regulation. For example, IP3 is crucial in calcium signalling through binding to receptors on the endoplasmic reticulum (ER) [11], while higher phosphorylated IPs (e.g. IP6, IP7 and IP8) regulate gene expression, DNA repair and RNA export, while further playing important roles in insulin signalling, phosphate homeostasis and cellular metabolism, which will be discussed in detail in the subsequent sections. Similarly, phosphoinositides have a wide range of functions in the regulation of actin cytoskeleton dynamics, endocytosis and exocytosis, ion channel activity and various signalling pathways, which will be discussed in this review. Dysregulation of phosphoinositide metabolism has been implicated in various diseases, including cancer [12,13], neurological disorders [14,15] and metabolic disorders [16,17]. Understanding the intricate roles of phosphoinositides and their regulatory mechanisms is essential for developing potential therapeutic interventions targeting these pathways.

### Phosphoinositides and IPs in metabolic liver disease

Metabolic dysfunction-associated steatotic liver disease (MASLD) is characterized by the accumulation of hepatic lipids exceeding 5% of liver weight and is commonly associated with obesity, hypertension, dysglycaemia and dyslipidaemia [18,19]. The global prevalence of MASLD is estimated to be approximately 38% [20], with a notably higher prevalence in specific population sub-groups, including 75% among people with overweight or obesity [21], 69% in those with type 2 diabetes (T2D) [22] and 69% in those with dyslipidaemia [23]. The advanced stages of MASLD include metabolic dysfunction-associated steatohepatitis (MASH), characterized by inflammation and hepatocyte ballooning, and liver fibrosis, which can further progress to cirrhosis and liver cancer, underlining the substantial healthcare burden of MASLD across global populations [20]. The limited FDA-approved treatment options for MASH and fibrosis, with Resmetirom (approved in March 2024) being the only drug on the market [24], highlight the need to better understand disease progression and to devise new therapeutic strategies.

The pathophysiology of MASLD is characterized by defective lipid metabolism, including increased fatty acid uptake, enhanced *de novo* lipogenesis (DNL) and impaired mitochondrial fatty acid oxidation [25], with the consequential lipid accumulation further leading to imbalanced hepatic lipoprotein metabolism [26]. Excessive lipid accumulation in the context of mitochondrial dysfunction contributes to an overproduction of reactive oxygen species (ROS) [27], endoplasmic reticulum stress and lipotoxicity [28–30]. Various enzymes involved in IP and phosphoinositide metabolism play important roles in hepatic metabolism and MASLD progression. For example, PI4P 5-kinase (*PIP5K1*) and phosphatidylinositol 4,5-bisphosphate (PIP2) are involved in the formation of cell-matrix adhesions in the liver, which contributes to tumour metastasis [31], while deletion of Pip4k2a and Pip4k2b (important in PIP2 synthesis) leads to hepatic steatosis due to defects in autophagy [32]. Furthermore, the PI remodelling enzyme membrane-bound O-acyltransferase 7 (MBOAT7) has been recently associated with severe liver disease, with loss of function promoting hepatic steatosis, inflammation and fibrosis [33]. In addition, targeting hepatic IPs has proven to be beneficial in advanced liver disease, for example with dietary phytic acid (IP6) supplementation attenuating oxidative stress and liver injury [34], improving fatty liver in rats [35] and mice [36], while further reducing liver metastasis of colorectal cancer [37], overall offering a rationale for studying the role of IPs and phosphoinositides in MASLD pathogenesis.

## Phosphoinositide and IP metabolism: Synthesis, uptake and recycling

### Extracellular uptake of myo-inositol

**Figure 1:**
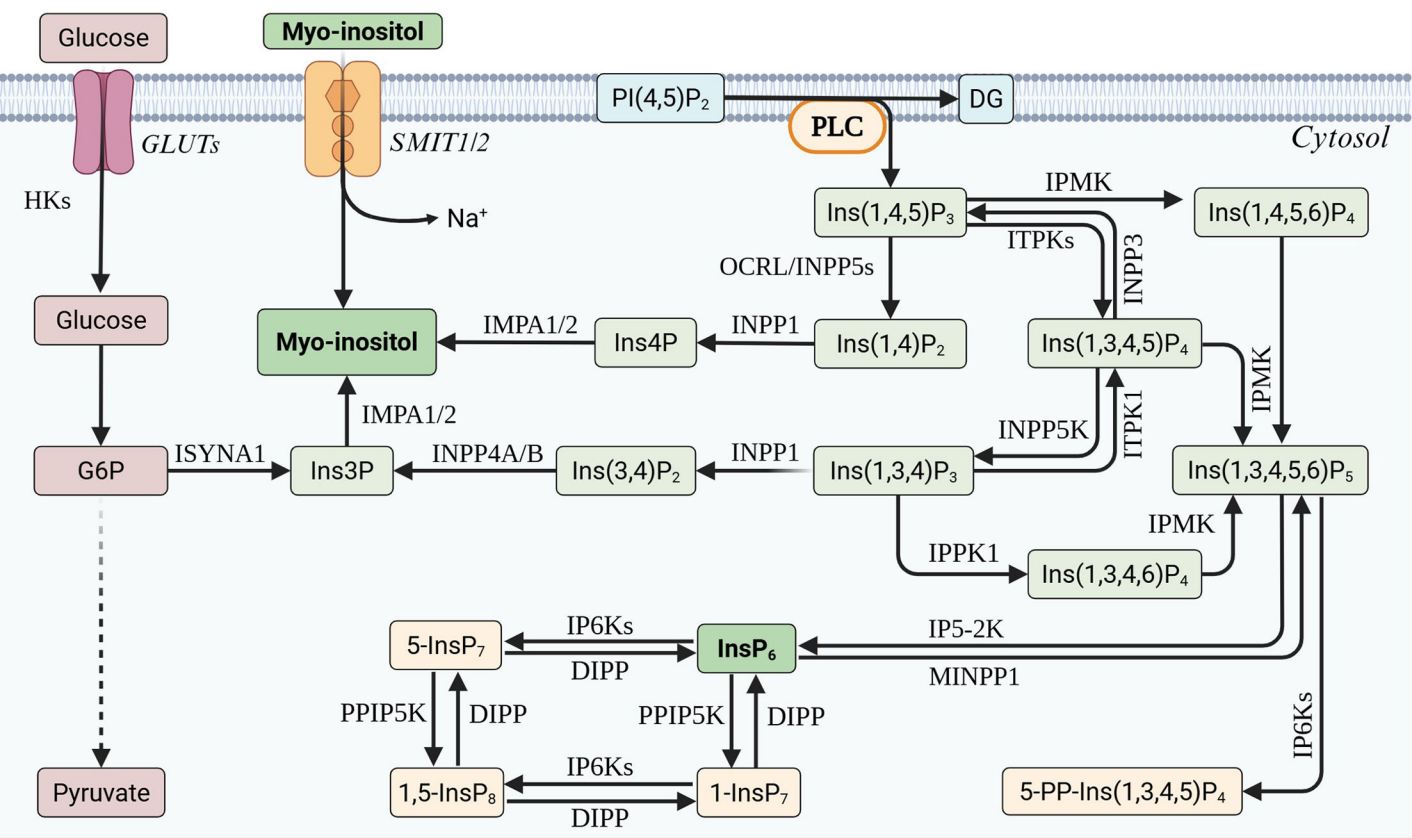
Biosynthesis of inositol phosphates (IPs) and pyrophosphates. IPs are synthesized via a lipid-dependent pathway from membrane-localized PI(4,5)P₂, which is hydrolysed by phospholipase C (PLC) to Ins(1,4,5)P₃. It can be sequentially dephosphorylated to myo-inositol or phosphorylated to generate higher-order IPs, including InsP₆ (i.e., IP6 or phytic acid) and inositol pyrophosphates. In parallel, myo-inositol can also be synthesized *de novo* from glucose-6-phosphate (G6P) via Ins(3)P. 1,5-InsP₈, 1,5-bisdiphosphoinositol tetrakisphosphate; 1-InsP₇, 1-diphosphoinositol pentakisphosphate; 5-InsP₇, 5-diphosphoinositol pentakisphosphate; 5-PP-Ins(1,3,4,5)P₄, 5-diphosphoinositol(1,3–5)tetrakisphosphate; DG, diacylglycerol; DIPP, diphosphoinositol polyphosphate phosphohydrolase; G6P, glucose 6-phosphate; GLUTs, glucose transporters; HKs, hexokinases; IMPA1/2, inositol monophosphatase 1/2; INPP1, inositol polyphosphate 1-phosphatase; INPP3, inositol polyphosphate 3-phosphatase; INPP4A/B, inositol polyphosphate 4-phosphatase A/B; INPP5K, inositol polyphosphate 5-phosphatase K; INPP5s, inositol polyphosphate 5-phosphatases; Ins(1,3,4)P₃, inositol 1,3,4-trisphosphate; Ins(1,3,4,5)P₄, inositol 1,3,4,5-tetrakisphosphate; Ins(1,3,4,5,6)P₅, inositol 1,3,4,5,6-pentakisphosphate; Ins(1,3,4,6)P₄, inositol 1,3,4,6-tetrakisphosphate; Ins(1,4)P₂, inositol 1,4-bisphosphate; Ins(1,4,5)P₃, inositol 1,4,5-trisphosphate; Ins(1,4,5,6)P₄, inositol 1,4,5,6-tetrakisphosphate; Ins(3,4)P₂, inositol 3,4-bisphosphate; Ins3P, inositol 3-phosphate; Ins4P, inositol 4-phosphate; InsP₆, inositol hexakisphosphate (phytic acid); IP5-2K, inositol pentakisphosphate 2-kinase; IP6Ks, inositol hexakisphosphate kinases; IPMK, inositol polyphosphate multikinase; IPPK1, inositol pentakisphosphate 2-kinase; ISYNA1, inositol-3-phosphate synthase 1; ITPK1, inositol-tetrakisphosphate 1-kinase; ITPKs, inositol-tetrakisphosphate kinases; MINPP1, multiple inositol polyphosphate phosphatase 1; OCRL, oculocerebrorenal syndrome of Lowe protein (inositol polyphosphate 5-phosphatase OCRL); PI(4,5)P₂, phosphatidylinositol 4,5-bisphosphate; PLC, phospholipase C; PPIP5K, diphosphoinositol pentakisphosphate kinase; SMIT1/2, sodium/myo-inositol cotransporter 1/2.

The cellular uptake of MI is primarily mediated by sodium-dependent MI transporters SMIT1 [38] and SMIT2 [39] (*SLC5A3* and *SLC5A11*) (Figure 1), as well as through the H+-MI transporter (HMIT or *SLC2A13*), which cotransports MI with H+ [40]. SMIT1 and SMIT2 are highly expressed in the kidney and to a lower extent in the liver [41,42], where they facilitate the efficient uptake of MI from the bloodstream, ensuring its availability for downstream metabolic processes. While SMIT1 has also been shown to transport glucose with low affinity [43], SMIT2, but not SMIT1, has the capacity to also import d-chiro-inositol [44]. The activity and/or expression of SMIT1 and SMIT2 is tightly regulated by hypotonic stress [45] and/or hormonal signals and metabolic cues. For example, SMIT1 expression is increased following acute high-glucose exposure and reduced in chronic hyperglycaemia [46], while on the other hand insulin has been suggested to enhance SMIT2-mediated inositol uptake [39], overall highlighting the interplay between glucose and inositol metabolism across insulin-sensitive tissues.

### *De novo* synthesis of myo-inositol

Alternatively, the kidneys and the brain are the primary sites of *de novo* MI synthesis from glucose-6-phosphate (G6P), derived from glucose via glycolysis. Inositol-3-phosphate synthase (*ISYNA1*) converts G6P to inositol-3-phosphate (Ins(3)P) in a NAD-dependent manner [47], which is one of the rate-limiting steps in IP synthesis and occurs in the cytosol. Subsequently, inositol-3-phosphate is dephosphorylated by inositol monophosphatases (*IMPA1* and *IMPA2*) to MI [48], ensuring a stable intracellular supply of MI even under fluctuating dietary conditions (Figure 1).

In HepG2 liver cells, ISYNA1 expression has been shown to be induced by glucose [49], suggesting that glucose exposure increases both inositol uptake (through SMIT1) and *de novo* inositol synthesis. On the other hand, ISYNA1 expression is inhibited by the mood stabilizer lithium [49], and, in this respect, the ability of lithium to deplete cellular inositol (particularly in the brain) has been proposed to explain the drug’s therapeutic action in treating bipolar disorder [50]. In addition, various studies have suggested that repression of *ISYNA1* (with accompanying reductions in MI generation) could serve as a common mechanism of carcinogenesis across tissues (e.g. 51,52), while on the other hand ectopic ISYNA1 expression increases MI content and suppresses tumour growth [52], which is likely related to ISYNA1 being a direct target of the tumour suppressor p53 [52] and induced by the cell-cycle regulator E2F1 [53]. Furthermore, ISYNA1 exhibits tissue-specific and gender-specific DNA methylation patterns [54], with inositol hexakisphosphate kinase 1 (IP6K1) [55], the enzyme that converts inositol pyrophosphate IP6 to IP7 (further discussed below), suggested to increase DNA methylation of the ISYNA1 promoter and to regulate ISYNA1 transcription [56]. These data highlight that alterations in methylation patterns could have a major impact on IP metabolism.

Similarly, lithium inhibits IMPA1 and IMPA2 activity, pointing to the broad impact of lithium on suppression of IP metabolism [57,58], with IMPA2 additionally identified as a susceptibility gene in bipolar disorder [59], schizophrenia [60] and febrile seizures [61]. A recent study published in *Cell Reports* further shows that the cleavage of a longer 3′ untranslated region (3′ UTR) of *Impa1* mediated by a protein complex containing the endonuclease argonaute 2 (Ago2) generates a shorter *Impa1* isoform that is stable, polyadenylated and necessary for maintaining axon integrity [62], highlighting the importance of IMPA1 and inositol metabolism in normal brain function.

While inositol monophosphatases have been extensively studied in neuropsychiatric disorders, emerging evidence has further linked IMPA1 and IMPA2 to the pathogenesis of various cancers. For example, IMPA1 and/or IMPA2 are either up- or down-regulated in breast, renal and ovarian cancer (among others, previously reviewed in [63]), and were shown to directly contribute to cancer growth by regulating Akt/mTOR signalling and epithelial‐mesenchymal transition [64–66]. Additionally, IMPA2 acts as an oncogene in cervical cancer through activation of the mitogen-activated protein kinase (MAPK) pathway [67]. IMPA2 expression has been shown to be suppressed by miR-25 in the setting of renal cancer, a micro RNA that is well known to be involved in the progression of many types of cancers [68], suggesting miRNA-regulated modulation of IMPA2 expression across various cancer settings. Given that IMPAs can dephosphorylate various targets, in addition to inositol-3-phosphate, including MI 1,3-diphosphate, MI 1,4-diphosphate, scyllo-IP, D-galactose 1-phosphate, glucose-1-phosphate, glucose-6-phosphate (among others) [48], future research is required to fully elucidate the role of the enzymes themselves and their major substrates/products in neuropsychiatric disorders and cancer development.

### Formation of phosphatidylinositol (PI)

**Figure 2:**
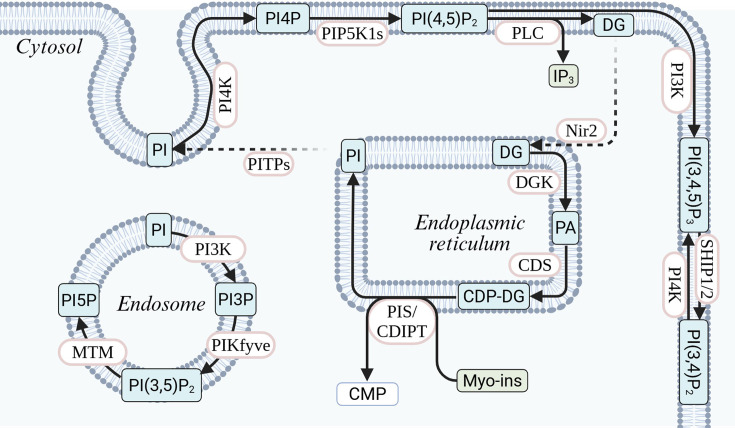
Synthesis and compartmentalization of phosphatidylinositol (PI) and phosphoinositides. PI is synthesized in the endoplasmic reticulum (ER) from myo-inositol and CDP-DG by PIS/CDIPT and transported to the plasma membrane via PITPs. At the plasma membrane, PI is phosphorylated to PI(4,5)P₂, which serves as a precursor for IP₃ and diacylglycerol (DG) synthesis through the enzymatic action of phospholipase C (PLC). DG is recycled back to generate phosphatidic acid and CDP-DG in the ER. At the endosome, PI is converted to minor phosphoinositides through sequential phosphorylation and dephosphorylation. CDP-DG, cytidine diphosphate-diacylglycerol; CDS, CDP-diacylglycerol synthase; CMP, cytidine monophosphate; DG, diacylglycerol; DGK, diacylglycerol kinase; IP₃, inositol 1,4,5-trisphosphate; MTM, myotubularin; myo-ins, myo-inositol; Nir2, phosphatidylinositol transfer protein Nir2; PA, phosphatidic acid; PI, phosphatidylinositol; PI(3,4)P₂, phosphatidylinositol 3,4-bisphosphate; PI(3,4,5)P₃, phosphatidylinositol 3,4,5-trisphosphate; PI(3,5)P₂, phosphatidylinositol 3,5-bisphosphate; PI(4,5)P₂, phosphatidylinositol 4,5-bisphosphate; PI3K, phosphoinositide 3-kinase; PI3P, phosphatidylinositol 3-phosphate; PI4K, phosphoinositide 4-kinase; PI4P, phosphatidylinositol 4-phosphate; PIKfyve, phosphoinositide kinase, FYVE-type zinc finger-containing; PIP5K1s, phosphatidylinositol-4-phosphate 5-kinases; PIS/CDIPT, phosphatidylinositol synthase / CDP-diacylglycerol-inositol 3-phosphatidyltransferase; PITPs, phosphatidylinositol transfer proteins; PLC, phospholipase C; SHIP1/2, SH2 domain-containing inositol 5-phosphatase 1/2.

To generate membrane-anchored phosphoinositides, MI is first attached to a lipid backbone. Specifically, the enzyme CDP-diacylglycerol–inositol 3-phosphatidyltransferase (*CDIPT*), also known as phosphatidylinositol synthase (PIS), uses CDP-diacylglycerol and MI as substrates generating CMP and phosphatidyl-1D-MI (i.e. phosphatidylinositol, PI) (Figure 2) [69]. Newly synthesised PI undergoes remodelling to acquire its characteristic fatty acid profile, with enrichment in stearic acid (C18:0) at the sn-1 position and arachidonic acid (C20:4) at the sn-2 position, and is embedded in the cytosolic leaflet of membranes, particularly the plasma membrane and the Golgi apparatus [69].

### Sequential phosphorylation of PI to form lipid-bound phosphoinositides

Membrane-bound PI can be phosphorylated at positions 3, 4 and/or 5 on the inositol ring to generate phosphoinositides, which act both as signalling molecules and as precursors for soluble IPs. These steps are catalysed by specific PI kinases and phosphatases, including the conversion of PI to PI(4)P (phosphatidylinositol-4-phosphate, commonly referred to as PIP) by PI 4-kinase (*PI4K*), the subsequent conversion of PIP to PI(4,5)P2 (phosphatidylinositol 4,5-bisphosphate, also referred to as PIP2) by PI4P 5-kinase (*PIP5K1*), and lastly the final conversion of PIP2 to PI(3,4,5)P3 (phosphatidylinositol(3,4,5)-trisphosphate, commonly referred to as PIP3) by Class I PI 3-kinase (*PIK3C*, more commonly known to as PI3K) [70] (Figure 2). PIP3 recruits PH domain-containing proteins to the membrane, including AKT [71] and PDK1 [72], thereby activating signalling cascades involved in cell growth, survival and proliferation. In this context, *PIK3CA*, the catalytic subunit of PI3K, is one of the most recurrently mutated genes in breast cancer [73] and cervical cancers [74], and PI3K inhibition has seen some success in pre-clinical and clinical trials, particularly as combination therapy with CDK4/6 [75], MEK [76] and PARP inhibitors [77].

In addition, PI3K/VPS34 can phosphorylate the inositol ring of PI at the 3-prime position, thereby generating PI(3)P (phosphatidylinositol-3-phosphate) on endosomes [78] or the cytosolic leaflet of cellular membranes [79]. Other variants like PI(3,5)P2 and PI(3,4)P2 are made via additional kinase activities. For instance, PI(3,5)P2 can be generated from PI(3)P through the PI 5-kinase PIKfyve/Fab1 [80], and further metabolized into PI(5)P through the myotubularin (MTM/MTMR) family of PI 3-phosphatases [81] (Figure 2).

The seven phosphoinositides mentioned above are generated by a total of 19 phosphoinositide kinases with complex multi-level regulation [82]. For example, PI4K and PIKfyve exist as large multi-protein megacomplexes of >700 kDa. PI4K is encoded by four genes (*PI4KA*, *PI4KB*, *PI4K2A* and *PI4K2B*), with the predominant pool of PIP at the plasma membrane generated by PI4KA [83]. PI4KA itself exists in the form of various multi-protein complexes, either containing PI4KA and two regulatory proteins (TTC7 and FAM126) [84], or PI4KA and the transmembrane protein TMEM150A and EFR3 [85], with all involved members having multiple, largely uncharacterized, phosphorylation sites. A recent study published in *Nature Communications* has shown that the calcineurin isoform CNAβ1, a protein phosphatase, is targeted to the PI4KA protein complex upon palmitoylation of its C-terminal tail, where it dephosphorylates FAM126A, promoting PI4KA complex formation and PIP production [86]. Increases in PI4KA and its activator EFR3A have been found in KRAS-driven pancreatic cancers [87], likely related to increases in PIP-mediated phosphatidylserine production required for oncogenic Ras clustering [88].

Similarly, PIKfyve functions as a large multi-protein complex containing two accessory proteins (Vac14 and Fig4 ), with intact complexes comprising one copy of PIKfyve and Fig4 and a pentameric Vac14 scaffold [89]. Within the protein complex, Fig4 is active both as a lipid phosphatase, reversing PIKfyve-mediated PI(3,5)P2 synthesis, and as a protein phosphatase targeting PIKfyve to stimulate its lipid kinase activity [89], with loss of Fig4 leading to reduced PI(3,5)P2 levels [90]. Recent evidence suggests that loss of function of the complex components is associated with neurological diseases, including amyotrophic lateral sclerosis (ALS) and primary lateral sclerosis [91], Charcot-Marie-Tooth disorder [90] [loss of Fig4] and paediatric neurological disorders [92] [loss of Vac14], while PIKfyve inhibition has been suggested to prevent ALS pathology [93], Alzheimer’s [94] and Parkinson’s Disease [95]. PIKfyve inhibitors have also shown promise in other disease models, including cancer [96] and viral infections [97].

### Generation of soluble IPs

In eukaryotes, the main source of soluble IPs is PI(4,5)P2 (i.e. PIP2), which is hydrolysed by phospholipase C (PLC) forming inositol 1,4,5-trisphosphate (IP3) and diacylglycerol (DAG) [98] (Figure 1). IP3 is soluble and diffuses through the cytosol to the ER, triggering calcium release (discussed in more detail below) [99]. Meanwhile, DAG remains in proximity to the cell surface, activating protein kinase C (PKC), which initiates additional cell signalling affecting cell growth, division and metabolism [100].

IP3 can undergo sequential dephosphorylation to regenerate MI. First, IP3 is dephosphorylated by inositol polyphosphate-5-phosphatase (INPP5F, gene name *OCRL*) to form inositol 1,4-bisphosphate (Ins(1,4)P2 or IP2) [101,102], which is further dephosphorylated by inositol polyphosphate-1-phosphatase (INPP1) to generate inositol 4-monophosphate (Ins(4)P), and finally by IMPA1/2 to regenerate free MI [103]. Alternatively, a fraction of IP3 is phosphorylated rather than degraded, fuelling the production of higher-order IPs. IP3 is phosphorylated by inositol 1,3,4-trisphosphate 5/6-kinase (ITPK1) to produce inositol 1,3,4,5-tetrakisphosphate (Ins(1,3,4,5)P4, also referred to as IP4) and by inositol polyphosphate multikinase (IPMK) to produce inositol 1,4,5,6-tetrakisphosphate (Ins(1,4,5,6)P4) [104]. IP4 can be dephosphorylated by INPP5 family phosphatases to regenerate IP3, a reaction favoured under physiological conditions [103,105,106]. Alternatively, IP4 can be dephosphorylated by INPP5K to form Ins(1,3,4)P3. Ins(1,3,4)P3 can then either be further dephosphorylated by INPP1 to generate inositol-3-phosphate (Ins (3)P), or phosphorylated by inositol 1,3,4-trisphosphate 5-kinase 1 (IPPK1) to produce another stereoisomer of IP4 [103,105,106]. IPMK acts as a major kinase hub by phosphorylating all IP4 species towards the production of inositol 1,3,4,5,6-pentakisphosphate (Ins(1,3,4,6)P5, also referred to as IP5), establishing a crucial branch point for IP metabolism. IP5 is further phosphorylated by inositol 1,3,4,5,6-pentakisphosphate 2-kinase (IP5-2K) to form inositol hexakisphosphate (IP6) (Figure 1).

Some soluble IPs can be synthesized directly from free MI by inositol kinases through the addition of phosphate groups to the inositol ring, though this is more common in plants and yeast than in mammals. The stepwise phosphorylation of MI can give rise to a broad spectrum of IP species, encompassing mono-, di-, tri-, tetra-, penta- and hexakisphosphates (IP6), including the ones detailed above, with >60 distinct IP isomers described to date [107]. Among these, the calcium-releasing factor IP3 is the most well-known, while the fully phosphorylated form IP6 is the most abundant IP metabolite in eukaryotic cells [108].

Notably, IP5 and IP6 can undergo further phosphorylation to yield inositol pyrophosphates (IPPs), which contain one or more high-energy diphosphate groups. Key examples include diphosphoinositol pentakisphosphate (IP7) and bis-diphosphoinositol tetrakisphosphate (IP8), both of which exhibit unique biochemical properties and signalling functions [109,110], which will be discussed in the subsequent sections. IP7 can be reversibly converted back to IP6 through the actions of diphosphoinositol polyphosphate phosphohydrolase (DIPP) phosphatases.

Distinct inositol kinases have been identified that add pyrophosphate groups to IP6 and other inositol phosphates, including IP6K (inositol hexakisphosphate kinase) [111] and PPIP5K (diphosphoinositol pentakisphosphate kinase) [112], a high-energy signalling molecule involved in the regulation of phosphate homeostasis, vesicle trafficking, DNA repair and cellular energetics [103,113]. In mammalian systems, IP6Ks (IP6K1/2/3) phosphorylate IP6 at the 5-position to produce 5-IP7 [114,115], whereas PPIP5Ks (PPIP5K1/2) phosphorylate the 1-position, generating 1-IP7 [116,117]. Given that 1-IP7 is a preferred substrate for DIPP, its cellular levels are typically lower than those of 5-IP7 [118,119].

## Crucial roles of inositol, phosphoinositides and IPs in cellular decision-making and metabolism

### Myo-inositol and D-chiro-inositol

In addition to being a starting point for the synthesis of PI and all phosphoinositides, and a building block for IPs and inositol pyrophosphates, free MI itself plays crucial roles in various cellular processes. For instance, MI can be transported through cellular membranes by the H^+^/MI transporter HMIT, which is abundant in the brain, adipose tissue and kidney, thereby acting as an osmolyte, helping cells manage water and ion balance [120]. Within the kidney, MI helps cells cope with high osmotic pressure during urine concentration [121], while within astrocytes, MI helps to maintain osmotic balance and protects neurons from volume changes due to ion fluxes [122]. This function is crucial in conditions like brain oedema or hyponatraemia, where cell volume regulation is critical. Therefore, it is not surprising that the levels of MI are substantially higher in the brain than in any other tissue [123], pointing to a critical role for inositol in normal brain function. Indeed, altered levels of MI have been observed in several neurological disorders, including depression [124], bipolar disorder [125] and Alzheimer’s disease [126], and MI supplementation has been studied as a natural mood stabiliser [127].

In addition to their role in the regulation of osmotic balance, inositol and its derivatives have important roles in glucose homeostasis and insulin signalling. In this respect, insulin-mimicking inositol phosphoglycans (IPGs) containing myo- or d-chiro-inositol (e.g. INS2 containing 3-o-methyl-d-chiro-inositol (d-pinitol)) promote glycogen synthesis in an insulin-dependent manner via activation of pyruvate dehydrogenase (PDH) [128]. INS2 has also been shown to stimulate protein phosphatase 2C (PP2C) activity [129], which is known to dephosphorylate and activate glycogen synthase [130], further enhancing glycogen synthesis. Given that PP2C activates PI3K [131] and inactivates AMP-activated protein kinase (AMPK) [132], INS2 is likely to play extensive roles in cellular energy homeostasis which require further investigation.

In addition to the role of INS2 in insulin action and energy homeostasis, d-chiro-inositol (DCI) itself may have more direct roles in insulin signalling. Upon insulin stimulation, MI is rapidly converted to DCI [133], with MI epimerization to DCI being severely impaired in the presence of insulin resistance in muscle, adipose tissue and liver [134]. Supplementation with DCI, but not MI, stimulates GLUT4 translocation and glucose uptake in myotubes and rat muscle *ex vivo* [135], while oral DCI administration in mice attenuates hepatic fatty acid uptake, reduces DAG levels and hepatic protein kinase Cε (PKCε) translocation and suppresses the expression of the gluconeogenic regulators *PCK1* and *G6P,* overall reducing hepatic gluconeogenesis and hepatic glucose output [136]. Furthermore, both DCI and MI were shown to increase the phosphorylation of Akt and ERK in human vascular endothelial cells (HUVEC) [137], while DCI and INS2 further increase autophosphorylation and internalization of the insulin receptor in primary hippocampal rat neurons [138]. In this context, supplementation with MI is successfully used in PCOS (polycystic ovary syndrome) to improve insulin sensitivity and ovulation [139].

The exact mechanism as to how free inositol participates in the insulin signalling pathway is currently not fully understood. It has been suggested that this may be through increased production of INS2, which allosterically modulates PP2C and subsequently activates PI3K [131], potentially contributing to GLUT4 translocation in an insulin-independent manner. However, this scenario does not take into account the direct action of inositol on insulin receptor autophosphorylation (which occurs upstream of PI3K). One study using molecular docking analysis of MI predicted active binding sites on PPARγ, GLUT4 and the insulin receptor [140], and future research is required to validate these findings and further elucidate the role of both MI and DCI within the insulin signalling pathway.

Lastly, there is an emerging role for free inositol in mitochondrial function and oxidative stress, particularly following an early study highlighting that the lithium-mediated suppression of IP metabolism is associated with up-regulation of mitochondria-related gene expression in the brain [141]. More recent evidence suggests that IMPA1 is localized mainly to mitochondria, indicating a potential role of IMPA1 and/or inositol in regulating mitochondrial function [142]. Indeed, the authors found that MI is capable of restricting AMPK-dependent mitochondrial fission by directly binding to AMPKγ and competing with AMP for AMPKγ binding [142]. Similarly, DCI supplementation in endothelial cells enhanced AMPK activity, suppressed mitochondrial fission through reductions in dynamin-related protein 1 (Drp1) phosphorylation, while further inhibiting NOX4 and enhancing Nrf2 activity [143], suggesting a role in oxidative stress. Improvements in antioxidant capacity and oxidative stress have been further shown in cultured adipocytes following DCI treatment [144]. This is supported by studies in C. elegans and Drosophila that showed that DCI enhanced antioxidant capacity, reduced oxidative stress and enhanced lifespan [145,146]. Dietary supplementation with a mixture of MI and d-chiro-inositol in diet-induced obese mice led to enhanced gene expression of markers of mitochondrial function and antioxidant defence in the heart, which was further associated with improvement in heart function [147]. Further mechanistic studies are required to fully elucidate how DCI, either directly or through its derivatives, affects mitochondrial capacity and oxidative stress.

### Phosphatidylinositol (PI)

PI performs a dual role in eukaryotic cells, both as structural membrane lipid and as precursor for phosphoinositide synthesis. Within cellular membranes, PI is a relatively minor lipid (5-10% of total lipids), with higher abundance in the brain [148], and can be found (at low levels) within virtually all intracellular membranes. PI is synthesized at the ER and delivered to other intracellular membranes by specific lipid transport proteins (LTPs) [149], with many LTPs transporting lipids at membrane contact sites (e.g. ER-cell membrane contact sites [150]). One such family of LTPs are phosphatidylinositol transfer proteins (PITPs) that bind and transport PI, with their primary role being the maintenance of cellular phosphoinositide levels [151]. Intracellular PI transport is required as the major PI kinases are localised to the plasma membrane, Golgi or endosomes, where PIP synthesis occurs [152]. In addition, the delivery of PI by PITPs to the Golgi induces localized changes within the lipid bilayer, that contribute to the budding of COPI-coated vesicles [153]. Furthermore, increased PI content within circulating very low-density lipoproteins (VLDL) impacts the net negative charge of lipoproteins and thereby increases cholesterol ester content within VLDL [154]. Similarly, PI was shown to stimulate reverse cholesterol transport by promoting the efflux of cholesterol from tissues to high-density lipoproteins (HDL) and thereby enhancing clearance of cholesterol through the liver, bile and faeces [154,155].

Within the plasma membrane, PI is primarily localized to the inner/cytoplasmic leaflet where it contributes to membrane asymmetry [156]. In addition, the inositol headgroup is relatively bulky, due to the hydration and hydrogen bond formation of the six hydroxyl groups of the inositol group, with the surface area of the headgroup being larger than the cross-sectional area of the acyl chains, which leads to a ‘lighter’ or less crowded packing of the acyl chains within the membrane and can cause increased water penetration within the lipid bilayer, as well as changes to membrane fluidity [157]. Given that transmembrane proteins comprise ~ 30% of the mammalian proteome [158], changes in the lipid microenvironment, including membrane fluidity, play an important role in metabolism, signalling and transport (reviewed in 159). Specifically, the fluidity (i.e. viscosity) of membranes determines the lateral diffusion of transmembrane proteins and influences protein-protein interactions that underlie cellular signalling. One of the best examples would be the impact of changes in fluidity of the inner mitochondrial membrane on the intramembrane diffusion of quinones, linking membrane fluidity with cellular respiration [160]. However, to our knowledge, little is known about how changes in PI content within cellular membranes affect the function of membrane proteins.

Lastly, PI is an important precursor for the formation of glycosylphosphatidylinositol (GPI) anchors on proteins, which are post-translational modifications covalently added to the carboxyl terminus of proteins. GPI anchors consist of a PI lipid, glycans comprising one glucosamine and three mannose sugars, and a phosphoethanolamine, and can be further modified through addition of glycan side-branches [161]. Instead of being embedded in the membrane via a transmembrane domain, these proteins are linked to the outer leaflet of the plasma membrane through the GPI anchor. To date, more than 150 proteins have been identified to contain GPI anchors, with a range of functions, including cell adhesion, signal transduction, immune response and specific enzymatic functions on the cell surface [162]. The physiological importance of GPI anchors is highlighted by the fact that loss-of-function mutations in genes required for GPI-anchor synthesis are embryonically lethal [163]. For an in-depth introduction into GPI physiology, which is beyond the scope of this review, please refer to a previous review [161].

### Lipid-bound phosphoinositides

**Figure 3:**
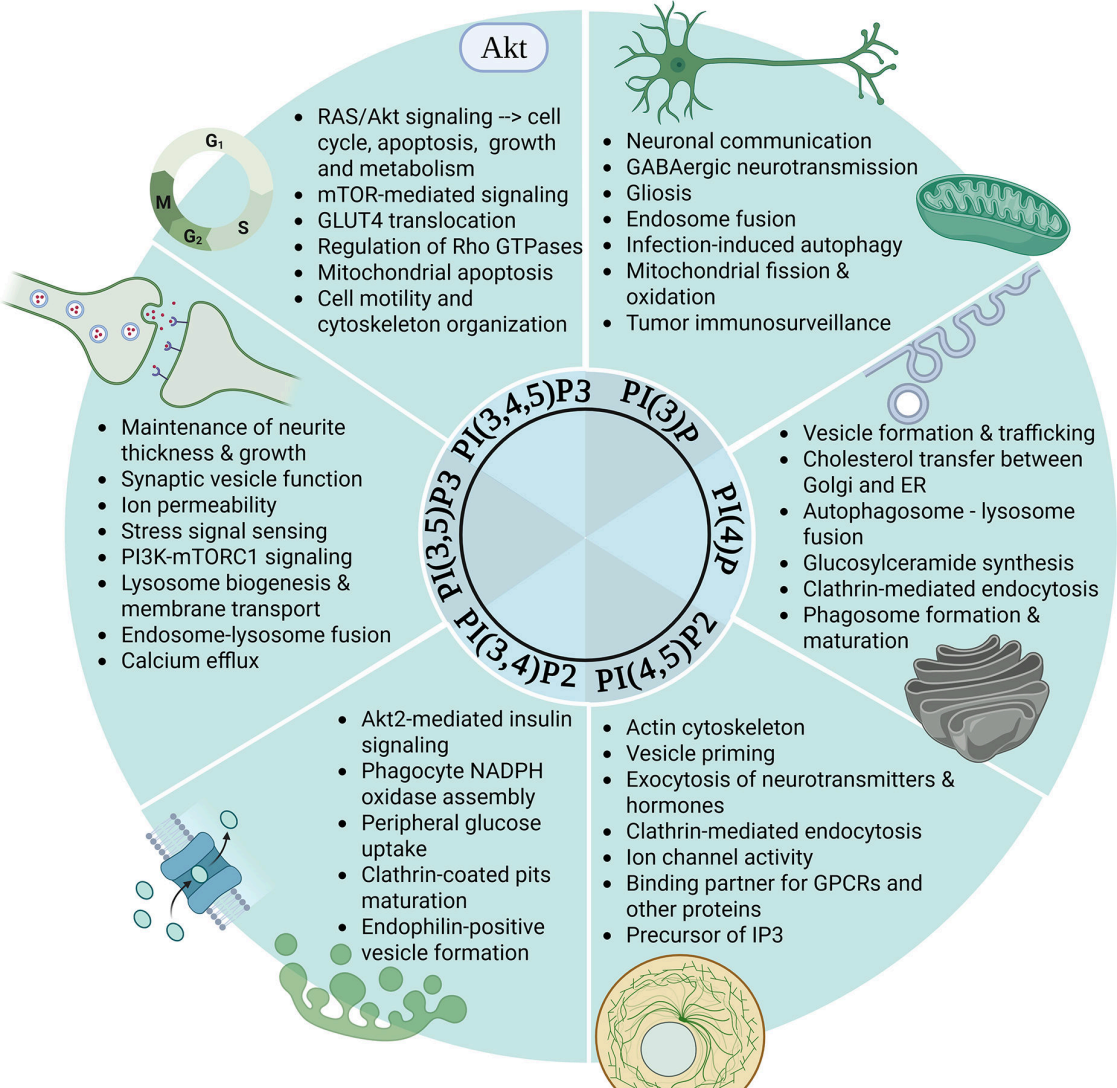
Physiological functions of the main phosphoinositides. Akt, protein kinase B; ER, endoplasmic reticulum; GABA, gamma-aminobutyric acid; IP₃, inositol 1,4,5-trisphosphate; mTOR, mechanistic target of rapamycin; mTORC1, mechanistic target of rapamycin complex 1; NADPH, nicotinamide adenine dinucleotide phosphate (reduced form); PI(3,4)P₂, phosphatidylinositol 3,4-bisphosphate; PI(3–,4,5)P₃, phosphatidylinositol 3,4,5-trisphosphate; PI(3,5)P₂, phosphatidylinositol 3,5-bisphosphate; PI(4,5)P₂, phosphatidylinositol 4,5-bisphosphate; PI3K, phosphoinositide 3-kinase; PI3P, phosphatidylinositol 3-phosphate; PI4P, phosphatidylinositol 4-phosphate; TGN, trans-Golgi network.

Phosphoinositides are localized to virtually any intracellular membrane, with the compartment-specific distribution of phosphoinositides leading to the selective recruitment of regulatory proteins to distinct subcellular localizations. In this respect, phosphoinositides can serve as a docking site for proteins with specific binding domains, including FYVE, PX, ENTH, CALM, PDZ, PTB and FERM domains, with pleckstrin homology (PH) domains being the most frequently found and studied [164]. More than 300 proteins contain one or even several PH domains with almost half of PH domain-containing proteins shown to bind phosphoinositides that direct proteins to their appropriate subcellular locations (165; reviewed in 166). However, having said that, specific phosphoinositide interactions for many PH domain-containing proteins are still unclear [167], and to date, only a small number of proteins has been shown to have specific affinity for a particular phosphoinositide [168,169], which will be discussed in more detail below (also summarized in Figure 3). The subsequent sections provide an overview of the primary physiological functions of the six main phosphoinositides.

#### Phosphatidylinositol-4-phosphate (PI(4)P or PIP)

PI(4)P (PIP) and PI(4,5)P2 (PIP2, discussed below) are the most abundant phosphoinositides in eukaryotes, consisting of about 0.5–1.5% of total phospholipids [170], with PIP being primarily localized to the inner leaflet of the plasma membrane [171] and the Golgi [172,173]. While for many years PIP has been suggested to be primarily a precursor for PIP2, recent studies have highlighted physiological roles for PIP that are distinct from PIP2 (and other phosphoinositides). PIP is highly enriched at the Golgi where it controls vesicular trafficking and membrane dynamics through a range of specific effector proteins. A role for PIP in vesicle formation within the Golgi was first described for its binding partner Sec14, a PI/phosphatidylcholine transfer protein that increases PIP content in the Golgi, thereby recruiting other effector proteins that promote the budding of exocytic vesicles from the Golgi [174]. Among others, these include the clathrin adaptor AP-1 complex [175] and the AP-interacting protein EpsinR [176], as well as recruitment of the clathrin adaptor Gga2p to the trans-Golgi network (TGN), enabling Golgi-to-endosome trafficking [177]. In addition, PIP recruits members of the four-phosphate-adaptor protein (FAPP) family of proteins to the TGN, which initiate membrane tubule formation and are essential for cargo transfer from the TGN to the plasma membrane [178].

In the past decade, several studies have further described a role for PIP in non-vesicular lipid transport. For example, a recent analysis of the cryo-electron microscopy structure of the TGN-localized P4-ATPase (Drs2p-Cdc50p) in yeast, a lipid flippase specific for phosphatidylserine and phosphatidylethanolamine transport, has identified a specific PIP-binding site on the P4-ATPase which relieves autoinhibition of the enzyme [179], highlighting a role for PIP in the generation and maintenance of membrane asymmetry. Furthermore, oxysterol-binding protein (OSBP)-related protein 5 (ORP5) and ORP8 interact with PIP through their respective PH domains, thereby tethering the ER to the plasma membrane and allowing for PIP-mediated transfer of phosphatidylserine from the ER to the plasma membrane [180,181]. Similarly, PIP was shown to be involved in the transport of cholesterol between the TGN and the ER by OSBP proteins [182], and specifically by ORP9 [183], with PIP markedly stimulating cholesterol transfer when present at the donor membrane (i.e. the TGN) [183]

Interestingly, PI4KIIα, the lipid kinase that generates PIP, acts as a binding partner for GABARAPs [184], proteins essential for autophagosome biogenesis and maturation, and is recruited from the Golgi to autophagosomes, where it generates PIP *in situ*, thereby exacerbating autophagosome-lysosome fusion and increasing autophagic flux [185]. This study nicely highlights that compartment-specific pools of phosphoinositides can also be generated through targeted recruitment of the specific PIP kinases to where they are needed within the cell.

In addition to vesicle formation and lipid transport, a second major role of PIP within the Golgi is the control of sphingolipid synthesis. For example, the ceramide transfer protein CERT extracts ceramide at the ER and is targeted to the Golgi through its PH domain, where it converts the bound ceramide to sphingomyelin and diacylglycerol [186]. A second fate of ceramide at the Golgi is the conversion to glucosylceramide, a precursor for complex glycosphingolipids that have key structural and signalling roles at the plasma membrane [187]. This process is facilitated by the glucosylceramide transfer protein FAPP2 which binds to PIP through its PH domain, thereby linking glycosphingolipid synthesis to PIP content within the Golgi [188].

In addition to having a striking role within the Golgi, PIP is synthesized on the inner leaflet of the plasma membrane, where it is involved in clathrin-mediated endocytosis, for example through interactions with auxilin 2 [189]. Furthermore, PIP functions in phagosome formation and maturation, accumulating in the forming phagocytic cup, thereby playing a role in the cellular engulfing and digestion of particulate matter, which is essential for the elimination of invading pathogens [190]. Similarly, PIP has been suggested to mediate phagosome–lysosome fusion [191], with this study being the first evidence for PIP (and not PIP2) as a central regulator of late phagosome maturation. However, having said that, the conversion of PIP to PIP2 on phagosomes is required for polymerization of F-actin [192], which stimulates membrane fusion [193], highlighting the tight interconnect between these phosphoinositide lipids.

#### Phosphatidylinositol 4,5-bisphosphate (PI(4,5)P2 or PIP2)

The physiological function of PIP2 can be sub-divided into its role as (i) a precursor for inositol 1,4,5-trisphosphate (IP3, discussed below) through the enzymatic action of PLC [98], (ii) a precursor for PIP3 through the enzymatic action of PI3K (also discussed below) [70] and (iii) as a primary messenger, which will be the focus of this section.

PIP2 is the main phosphoinositide localized to the plasma membrane, comprising about 1% of the phospholipids within the cytoplasmic leaflet, and can interact with a multitude of effector proteins through their respective PIP2-binding domains, including PH, ANTH, ENTH, C2, FERM, PDZ and Tubby domains (reviewed in 194). Furthermore, with PIP2 being negatively charged, it can further bind a range of other proteins, such as ion channels and receptors [195]. Due to the large number of roles of PIP2 and the numerous binding partners, here we will only focus on some major examples (for a more complete list, please refer to [194]).

The first PIP2-binding partner was described in 1985. Profilin, as well as the profilactin complex (profilin/actin complex), was shown to bind to PIP2 causing rapid actin polymerization [196]. This finding was followed by a study in 1987, which highlighted that PIP2 inhibits the actin filament-severing properties of gelsolin [197], suggesting a role for PIP2 in the regulation of the actin cytoskeleton. The cytoskeleton is important in cell rigidity and is attached to the plasma membrane by a range of ERM proteins, including Ezrin, Radixin and Moesin (abbreviated as ERM), which have a high affinity to PIP2 through their FERM domains [198]. Similarly, class I myosins are recruited to the plasma membrane by PIP2 through their PH domains and form linkers between the membrane and the actin cortex, contributing to actin motility [199].

In addition, PIP2 is required for exocytosis on the plasma membrane, i.e. the release of hormones and neurotransmitters into the extracellular space, with high concentrations of PIP2 found on the plasma membrane of neuroendocrine cells [200]. There, PIP2 serves to recruit and/or activate multifunctional effector proteins (e.g. CAPS [201], syntaxin-1 [202], the calcium-sensor synaptotagmin-1 and the vesicle priming protein Munc13-2 [203]) that regulate SNARE function required for vesicle priming and exocytosis [200]. In addition to a prominent role in exocytosis, PIP2 is important in clathrin-mediated endocytosis by localising the required endocytic machinery to the site of endocytosis. Specifically, various clathrin adaptor proteins (e.g. the AP2 complex [204], Epsin [205], dynamin [206], β-arrestin [207], among others) bind to PIP2, subsequently allowing for the recruitment of Clathrin to the plasma membrane to initiate the formation of clathrin-coated pits.

As detailed above, PIP2 can also bind ion channels and ion transporters on the plasma membrane largely due to its highly negative charge, with the first paper pointing to this function for PIP2 in cardiac Na+, Ca_2_+exchange in 1996 [208], with more than 30 ion channels and transporters reported to date (reviewed in 209,210), including a role for PIP2 in current amplitude and voltage sensitivity of the KCNQ2 channel [211]. Lastly, PIP2 was shown to bind to a number of GPCRs, with the first study in 2018 identifying key residues on G proteins through which PIP2 forms bridging interactions with GPCRs, including the β1 adrenergic receptor [212]. In addition, PIP was shown to form a bridge between the neurotensin receptor 1 (NTSR1) and the β-arrestin [207]. Taken together, these studies highlight the broad range of physiological roles for PIP2 across many tissues, and therefore it is not surprising that dysregulation of PIP2 and/or its vast variety of effector proteins has been implicated in many pathophysiological conditions, including Alzheimer’s [213], Crohn’s disease [214], atherosclerosis [215] and cancer [216], among others.

#### Phosphatidylinositol (3,4,5)-trisphosphate (PI(3,4,5)P3 or PIP3)

PIP3, in combination with its kinase PI3K (reviewed elsewhere [217]), has been considered as one of the most important phosphoinositides, regulating a myriad of cellular functions, including proliferation, cytoskeleton arrangement, vesicle trafficking and cell metabolism. PIP3 is primarily found in the inner leaflet of the plasma membrane, where it serves as a docking site for proteins that primarily contain a PH domain, thereby activating and propagating cellular signalling cascades (reviewed in 218). Of the 300 + PH-domain containing proteins that have been identified to date, about 40 have been shown to bind to PIP3.

The most well-known target of PIP3 is protein kinase B/Akt, with the binding to PIP3 through its PH domain leading to Akt phosphorylation (at residues Thr308 and Ser473) and activation [219], permitting substrate binding and restricting Akt activity to the plasma membrane [220]. Specifically, Akt binding to PIP3 increases Akt phosphorylation by phosphoinositide-dependent kinase 1 (PDK1) at Thr308 and by mTOR Complex 2 (mTORC2) at Ser473, leading to full Akt activation and substrate binding [221]. In addition, PIP3 binding to Akt promotes Akt methylation and ubiquitination, which in turn sustains AKT phosphorylation [222,223]. PDK1 itself is recruited to the plasma membrane by PIP3, with mutations of the PH domain of PDK1 suppressing full activation of Akt [224]. On the other hand, acetylation of Akt and PDK1, an effect reversed by the deacetylase SIRT1, blocks binding to PIP3, thereby preventing membrane localization and Akt phosphorylation [225], overall highlighting that post-translational modifications of Akt (including methylation, ubiquitination and acetylation) are important in synergizing with PI3K signalling to control Akt activation. Furthermore, it has been shown that PI3K-AKT signalling depends on PI3Kα and PI3Kβ, while PI3Kδ and PI3Kγ have been suggested to be largely dispensable, with PI3Kα being the major mediator of RAS-mediated stimulation of Akt phosphorylation [226,227], an effect that is not specifically mediated by p110α, the catalytic subunit of PI3Kα [227] or the ubiquitous p110β isoform [228]. In contrast, RAC1 and CDC42 from the RHO subfamily of small GTPases have been shown to bind and activate p110β via its RAS-binding domain [228].

Excessive PIP3 at the plasma membrane can lead to hyperactivation of Akt, suppression of apoptosis and increased cell proliferation, commonly observed in 50% of human cancers [229]. In addition, a mutation within the PH domain of Akt (E17K), reported in various cancer settings, shows stronger Akt binding to PIP3 and increased PDK1 phosphorylation, contributing to cell proliferation in cancer [230]. On the contrary, mutations within the PH domain of Akt [231] and/or PDK1 [224] can disrupt its ability to bind to PIP3, thereby suppressing its activation and downstream signalling, and driving the development of insulin resistance [232].

The PIP3-mediated activation of Akt has various tissue-dependent cellular roles, including cell cycle progression, apoptosis, cellular growth and metabolism. Overall, more than 100 Akt substrates have been reported that are involved in a myriad of biological pathways [221]. Here we summarize some of the major cellular changes that occur upon PIP3-mediated Akt activation. Following PIP3 binding, activated Akt can translocate to the nucleus where it promotes the nuclear release of Forkhead box O (FoxO) transcription factors and inhibits the transcriptional function of FoxO, thereby contributing to transcriptional regulation of cell cycle progression, apoptosis, autophagy and metabolism [233]. Similarly, Akt inhibits glycogen synthase kinase 3β (GSK3β), which promotes cyclin D1 activity and further contributes to cell cycle progression [234]. Additionally, activated Akt has the capacity to phosphorylate the death receptor BCL2 associated agonist of cell death (BAD), inhibiting its translocation to mitochondria, and subsequently leading to the inhibition of mitochondrial apoptosis [235], while phosphorylating and activating inhibitory-κB kinase (IKK), ultimately leading to the translocation of the transcription factor NF-κB to the nucleus, where it triggers an anti-apoptotic transcriptional program [236]. In the context of cellular growth, PIP3-activated Akt phosphorylates and inhibits tuberin, a component of the tuberous sclerosis complex (TSC), which leads to the release of mTOR suppression [237], and an increase in mTOR-mediated signalling events that regulate cell growth and protein synthesis [238], as well as various aspects of mitochondrial function [239] and lipid metabolism [240]. Lastly, Akt has a well characterized role in insulin sensitivity and glucose metabolism, on one hand inhibiting hepatic glucose output, while on the other hand exacerbating glucose transporter (GLUT)-mediated glucose uptake in peripheral tissues [241].

Beyond its function in Akt signalling, a role for PIP3 has been described in the regulation of Rho GTPases, small G proteins that regulate cell cycle and cytoskeleton organization, with PIP3 specifically binding to guanine nucleotide exchange factors (GEFs) that regulate the activity of Rho GTPases [242]. For example, PIP3-dependent Rac exchanger 1 (P-Rex1) is a GEF that is activated by binding to PIP3 [243] and responsible for nucleotide exchange to control cell motility and morphology, as well as chemotaxis [244]. Similarly, PIP3 stimulates the activity of Grp1 (general receptor for 3-phosphoinositides 1) family GEFs that regulate membrane trafficking and actin dynamics [245], and Vav, a GEF that is responsible for mitogen stimulation of cytoskeletal changes [246]. Importantly, Grp1 is also a guanine nucleotide exchange factor for ADP-ribosylation factor 6 (ARF6) which promotes GLUT4 vesicle formation and GLUT4 recycling [247], highlighting the complex nature of PIP3-mediated regulation of insulin signalling and glucose metabolism beyond Akt.

#### Phosphatidylinositol 3,4-bisphosphate (PI(3,4)P2)

While historically PI(3,4)P2 has been considered an inconsequential byproduct of PIP3 hydrolysis by SHIP phosphatases [248], and despite most studies that investigate phosphoinositide physiology not distinguishing the functional contributions of PIP3 and PI(3,4)P2 (given that both can be synthesized by PI3K), recent evidence suggests that PI(3,4)P2 plays a distinct role in insulin signalling, endocytosis and cell migration, as well as neuronal dynamics. In this respect, quantitative imaging techniques have suggested distinct spatiotemporal distribution of PIP3 and PI(3,4)P_2_, particularly in response to growth factor stimulation, which allows for isoform-specific Akt recruitment and activation [249]. Specifically, PI(3,4)P2 shows higher affinity to Akt2 at both the plasma membrane and early endosomes, whereas Akt1 and Akt3 preferentially bind to PIP3 exclusively at the plasma membrane [249]. Interestingly, insulin stimulation leads to PI(3,4)P2 accumulation [250], which points to an insulin receptor/PI(3,4)P2 specific activation of Akt2. At the plasma membrane, PI(3,4)P2 binding to Akt2 leads to a conformational change in Akt2, exposing the activation loop and making it more accessible for phosphorylation by PDK1 [251]. Akt2 is highly expressed in insulin-responsive tissues (e.g. skeletal muscle, adipose tissue and liver), where it is primarily involved in the regulation of insulin signalling and glucose metabolism [252], pointing to a crucial role for PI(3,4)P_2_ in the maintenance of systemic glycaemic control.

Only a few proteins were shown to selectively bind PI(3,4)P2 and no other phosphoinositide within the plasma membrane. Among those are the tandem PH domain containing proteins TAPP1 and TAPP2 that are recruited to the plasma membrane by PI(3,4)P2 [253], leading to suppression of B-cell and Akt activation [254]. Knock-in mice that express mutant TAPP1 and TAPP2 incapable of binding PI(3,4)P2 exhibit enhanced Akt activation, improved insulin sensitivity and increased glucose uptake into skeletal muscle [255], highlighting that TAPP1 and TAPP2 act as negative regulators of the insulin signalling pathway. In addition, the Phox homology (PX) domain on effector proteins, named after p40phox subunit of the NADPH oxidase complex, targets proteins to membranes through phosphoinositide binding, with the PX domain of p47phox (also referred to as neutrophil cytosol factor 1) preferably binding PI(3,4)P_2_ [256] and playing a pivotal role in the assembly of phagocyte NADPH oxidase [257].

In addition to PIP and PIP2 playing a role in clathrin-mediated endocytosis, a similar role has been identified for PI(3,4)P2. Specifically, PI(3,4)P2 is required for the selective enrichment of the BAR domain proteins SNX9 [258,259] and SNX18 [259] at late-stage endocytic intermediates, which is essential for the maturation of clathrin-coated pits before fission. Depletion of PI(3,4)P2 inhibits the maturation of clathrin-coated pits [258]. Furthermore, it has been shown that PI(3,4)P_2_ is enriched within the leading edge of migrating cells where it binds lamellipodin, followed by lamellipodin-mediated recruitment of the membrane-deforming protein endophilin, leading to the formation of endophilin-positive vesicles that aid in receptor internalization into tubular endosomes [260,261].

PI(3,4)P2 recruitment of SNX9 has been further shown to play a role in the apical–basal polarization of epithelial cells. Specifically, PI(3,4)P2 is enriched within the apical membrane, where SNX9 binding leads to the formation of an apical domain and contributes to epithelial polarity [262]. In addition, PI(3,4)P2 was found to be enriched in membrane ruffles, which are highly dynamic actin-based membrane structures that are involved in pinocytosis, suggesting a role for PI(3,4)P2 in dorsal ruffle formation [263]. A recent study has further suggested a role for PI(3,4)P2 in membrane-scission machinery within the lens through recruitment of the ESCRT-II component (Vps36), a process that protects from senescence and the early onset of cataracts [264].

Recently, a novel role has been described for PI(3,4)P2 locally synthesized within late endosomes/lysosomes, where it triggers recruitment of inhibitory 14-3-3 proteins to lysosomes. Lysosomal 14-3-3 proteins associate with the Raptor subunit of mTORC1 and thereby repress lysosomal mTORC1 activity [265]. Lysosomal repression of mTORC1 was further shown to be facilitated by ORP1L-mediated export of cholesterol from lysosomes [266], with cholesterol contributing to mTORC1 recruitment and activation at the lysosomal surface [267]. Apart from 14-3-3 and ORP1L, other effector proteins of lysosomal PI(3,4)P2 remain largely unknown.

Two PI(3,4)P2-specific phosphatases have been identified, type I and II inositol polyphosphate-4-phosphatase (INPP4A and INPP4B) [258,268], producing PI(3)P (discussed in the next section), with both isoforms being ubiquitously expressed [269]. Interestingly, deletion of INPP4A in mice leads to neurodegeneration and involuntary movement disorders, with the authors showing that INPP4A protects neurons from excitotoxic cell death [270]. INPP4A was further shown to dampen airway inflammation and alleviate asthma in mice [271], with a single nucleotide polymorphism (SNP) in INPP4 correlating with asthma susceptibility in patients [272], which has been contributed to an INPP4-mediated reduction in oxidative stress-induced PI(3,4)P2 accumulation [273]. In contrast, INPP4B deficiency in mice led to defects in bone homeostasis, with mice showing increased osteoclast differentiation, decreased bone mass and osteoporosis [274]. Furthermore, loss of INPP4B has been associated with enhanced cell proliferation and poor prognosis in many cancers (reviewed in 273). Future studies are required to elucidate if the above pathophysiological effects are related to changes in INPP4 expression/activity or to changes in the substrates and products of INPP4’s enzymatic actions, including changes in PI(3,4)P2 content.

#### Phosphatidylinositol 3,5-bisphosphate (PI(3,5)P2)

**Figure 4:**
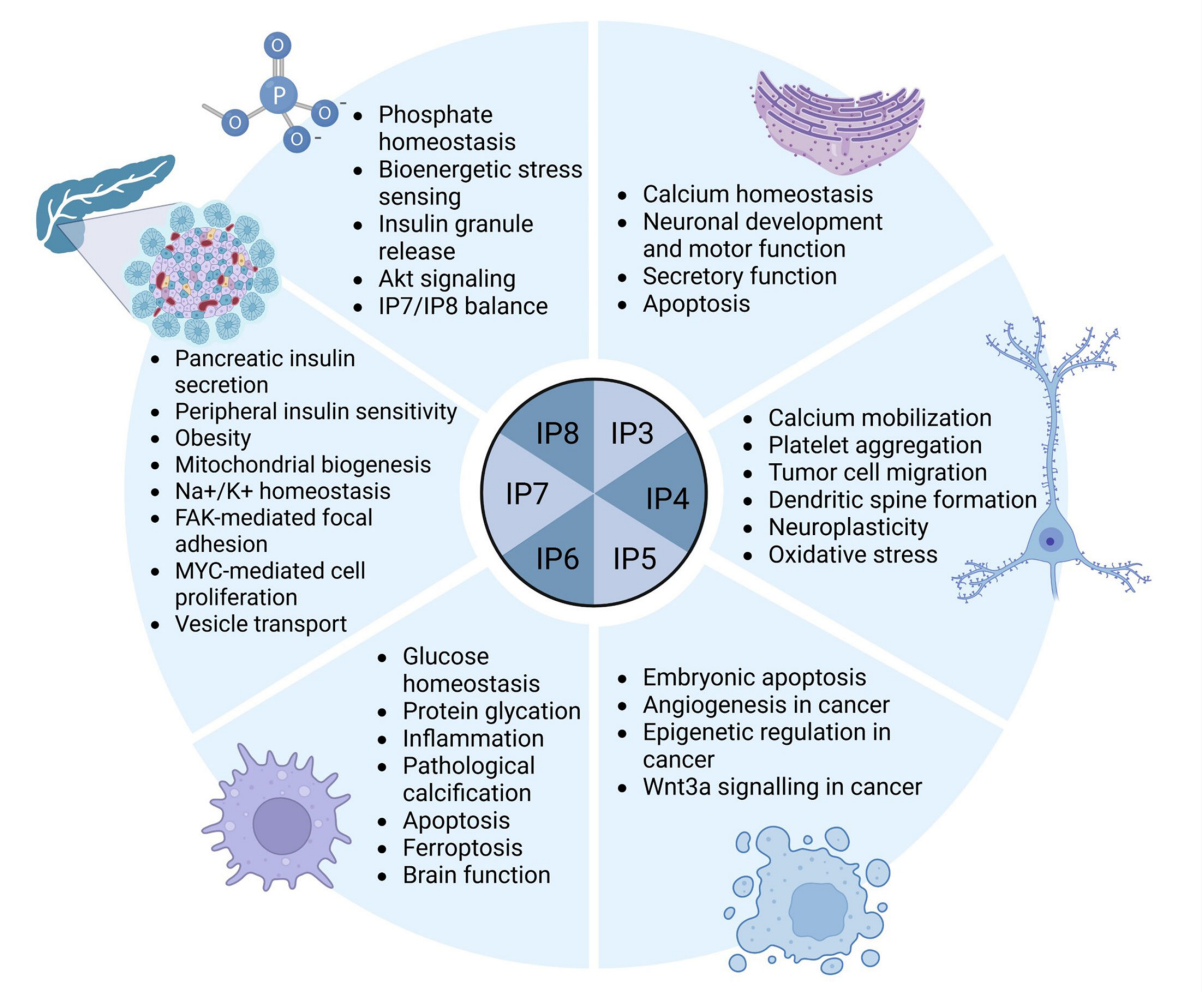
Physiological functions of the main inositol phosphates. Akt, protein kinase B; FAK, focal adhesion kinase; IP₃, inositol 1,4,5-trisphosphate; IP₄, inositol 1,3,4,5-tetrakisphosphate; IP₅, inositol 1,3,4,5,6-pentakisphosphate; IP₆, inositol hexakisphosphate; IP₇, diphosphoinositol pentakisphosphate; IP₈, bisdiphosphoinositol tetrakisphosphate; MYC, v-myc avian myelocytomatosis viral oncogene homolog; Wnt3a, wingless-type MMTV integration site family member 3 alpha.

PI(3,5)P2 has low abundance when compared with other phosphoinositides, comprising only about 0.05–0.1% of total PI lipids. In mammals, PI(3,5)P2 is synthesized from PI(3)P (discussed in the next section) by a complex consisting of the lipid kinase Fab1/PIKfyve, the scaffolding protein Vac14 and the lipid phosphatase Fig4 [275]. Deletions/inactivation of either of these proteins in mice leads to embryonic lethality and neurodegeneration [90,275–277], highlighting the importance of PI(3,5)P2 in development and normal cellular functions, particularly in the nervous system. Indeed, the neurological disorders Charcot-Marie-Tooth syndrome, amyotrophic lateral sclerosis and primary lateral sclerosis have been associated with reductions in PI(3,5)P2 content [90,91]. Supporting a role in brain function, recent studies have shown that PI(3,5)P2 is involved in axonal delivery of synaptic vesicle and active zone proteins to developing synapses [278], and in the maintenance of neurite thickness mediated by NSG1/NEEP21, a neuron-specific endosomal protein [279]. Interestingly, PI(3,5)P2 encapsulated within nanoparticles leads to neuroprotection, including alleviation of motor neuron loss in pre-clinical models of ALS, which has been associated with activation of mucolipin transient receptor potential 1 (TRPML1) channels [280]. Supporting this notion, PI(3,5)P2 has been shown to modulate ion permeability by binding to and activating endolysosome-localized TRPML channels [281], and through activation of the two-pore channel (TPC) under conditions of depolarizing membrane potential [282].

A recent study suggested that PI(3,5)P2 specifically generated at the lysosomes terminates Class I PI3K activity via specific interaction with its regulatory p85 subunit, with this process suppressing neurite growth [283]. In addition, activity of lysosomal mTORC1, an important regulator of cell growth, is dependent on PIKfyve and PI(3,5)P2 content, with the mTORC1 component Raptor directly interacting with PI(3,5)P2 [284]. On the other hand, the tuberous sclerosis protein complex (TSC complex), a key mediator of metabolic signals, is also recruited to lysosomes through binding to PI(3,5)P2, where it inhibits mTORC1 and thereby integrates nutrient and stress signals into an orchestrated cellular response [285]. Overall, these studies suggest that PI(3,4)P2 plays a crucial role in the PI3K-mTORC1 signalling network, particularly at the lysosomal level, and directs cellular metabolism and cellular growth.

In addition to a prominent role in the nervous system, TRPML1, bound to PI(3,5)P2 within late endosomal and lysosomal membranes, has multiple roles in lysosome biogenesis, endosome-lysosome fusion, exocytosis and lysosomal membrane transport (reviewed in 286). For example, TRPML1 promotes recruitment of syntaxin 7 (Stx7) and vesicle-associated membrane protein 7 (VAMP7) to lysosomes, driving calcium efflux and leading to local vesicle fusion events [287]. Similar to the other phosphoinositides, PI(3,5)P2 is also involved in the regulation of membrane trafficking and protein sorting, particularly the retrograde traffic from early endosomes to the TGN through SNX1 and SNX2 [288,289], and lysosomal degradation through binding to SNX11 [290], while SNX10 has been shown to regulate the delivery of PI(3)P, the precursor of PI(3,5)P2, from endocytic compartments to (phago)lysosomes [291].

#### Phosphatidylinositol-3-phosphate (PI(3)P)

PI(3)P plays a crucial role in a variety of cellular processes, including autophagy and membrane trafficking, and is primarily localized to endosomal membranes and the autophagosome machinery, with the class III PI 3-kinase, VPS34, being the main PI(3)P-synthesizing enzyme on endosomes [292]. Within endosomal membranes, particularly in the brain, PI(3)P binds several effector proteins, including activity-regulated cytoskeleton-associated protein (Arc) which serves as a pivotal regulator for neuronal communication [293] and gephyrin which affects GABAergic neurotransmission [294]. Loss of neuronal PI(3)P results in extensive gliosis and progressive neurodegeneration [295], potentially as a consequence of perturbed synaptic vesicle endocytosis [78].

In non-neuronal cells, PI(3)K plays crucial roles in endosome fusion through a range of functions, including binding of early-endosomal autoantigen EEA1 and regulation of Rab5-dependent endocytic transport [296], surface delivery of endosomal cargo [297], directional transport of recycling cargo from early endosomes to the endocytic recycling compartment [298], endosomal activation for initiation of autophagy during viral infection through sorting nexin 5 (SNX5) [299], as well as increased contact formation between tubular ER membranes and early endosomes, which was mediated by binding of the related ER proteins RRBP1 and kinectin 1 to PI(3)P on endosomes [300]. Importantly, this PI(3)P-induced reshaping of the ER has been shown to suppress mitochondrial fission and promote the formation of a hyperfused mitochondrial network, leading to a defect in mitochondrial fatty acid delivery and impairment in mitochondrial oxidative metabolism [300]. These data highlight a crucial role for PI(3)P in endosomal lipid signalling and mitochondrial function.

While PI(3)P has also been shown to be localized to mitochondria [301], to our knowledge, the role of mitochondria-localized PI(3)P is unclear. Similarly, while a link has been established between endosomal PI(3)P and mitochondrial oxidative capacity [300], little is known about the direct role of compartment-specific PI(3)P pools on mitochondrial function. In contrast, the role of PI(3)P in autophagy is better understood [302], with the WD-repeat protein interacting with phosphoinositides (WIPI) family of proteins being important PI(3)P effectors bringing together beclin 1, p150 (also known as PIK3R4) and ATG14L, among other components, for effective autophagosome formation [303]. Interestingly, a proximity-based screening approach has recently identified WIPI2 (which binds PI(3)P) in proximity to mitophagy receptors [304], and WIPI2 was subsequently shown to recruit valosin containing protein (VCP) to mitochondria in response to mitochondrial damage [305]. In addition, a recent study suggested that PI(3)P, and more specifically the PI(3)P phosphatase Ymr1, might be involved in mitophagy [306]. However, further research is required to fully elucidate the direct role of PI(3)P in mitophagy and mitochondrial energy metabolism.

In addition, previous research has described a role for PI(3)P in cell division, by localizing to the midbody and recruiting the cytokinesis regulatory machinery [307], in toll-like receptor 9 (TLR9)-induced IgA immunity [308], in COPII-mediated ER-to-Golgi trafficking of STING, which is essential for control of infections and for tumour immunosurveillance [309], and in survival of rod photoreceptor cells in the eye [310].

### Soluble inositol phosphates

IPs are versatile small molecule messengers that play crucial roles in various aspects of cellular decision-making, including a range of signalling pathways related to apoptosis, cell growth, DNA repair and calcium homeostasis, among other functions. While this group of inositol metabolites has more potential members than the lipid-bound phosphoinositides, only a small subgroup has been well-studied, with the major isoforms discussed below (also summarized in Figure 4).

#### Inositol 1,4,5-trisphosphate (IP3)

Calcium is a universal intracellular messenger in mammalian cells that plays important roles in cell division, apoptosis, gene expression, muscle contraction, among many other functions. IP3, primarily synthesized following PIP2 hydrolysis, is a common contributor underpinning these processes by regulating intracellular calcium concentrations. Specifically, IP3 binds to calcium-permeable IP3 receptors that are primarily localized to the ER (reviewed in 311,312), releasing calcium into the cytosol. In addition, IP3 receptors have been found to be localized to ER-mitochondria contact sites that couple cellular calcium homeostasis and mitochondrial function [313], a process regulated by Kras-induced actin-interacting protein (KRAP, also known as ITPRID2) [314].

Three IP3 receptors have been described in mammals (IP3R1, IP3R2 and IP3R3), with the sub-types having the capacity to form either homo- or hetero-tetramers. Interestingly, with a size of approximately 1.2MDa, they represent some of the largest ion channels currently known. IP3R1 is the predominant IP3 receptor in the brain, playing an essential role in ataxia and motor learning, with IP3R1 deficient mice showing seizures and dying *in utero* or by weaning age [315], whereas the other two subtypes are expressed (and frequently co-expressed) across many peripheral tissues [316]. While deletion of either IP3R2 or IP3R3 does not lead to any major developmental or phenotypic differences, double knockout mice show severe defects in the secretory function of many tissues, including the pancreas and salivary glands, and show gastrointestinal defects [317].

IP3 binding to the IP3 receptors is regulated at multiple levels, with the IP3 receptors containing various consensus sites for phosphorylation and many docking sites for protein kinases and phosphatases (reviewed in 318). For instance, binding of calmodulin [319] and IRBIT [320] competes with the IP3 binding site, suppresses receptor activity and IP3-mediated calcium release. In addition, B-cell lymphoma-2 (Bcl2), an important regulator of apoptosis, has been identified as a docking protein for calcineurin on the IP3 receptor [321], leading to a decrease in calcium within the ER. Bcl2 binding to IP3R1 is suppressed by Bax/Bak, leading to a reduction in IP3R1 phosphorylation and reduced calcium leak from the ER [322]. Furthermore, cyclic AMP-dependent protein kinase A (PKA) can phosphorylate two distinct sites on IP3R1 [323], which leads to an increase in the sensitivity of IP3R1 towards IP3, an effect counteracted by protein phosphatase 1α (PP1α) [324]. Similarly, cyclic GMP-dependent protein kinase (PKG) was shown to phosphorylate IP3R1 at the same sites as PKA [325,326]. PKA- and/or PKG-mediated phosphorylation is further likely to affect the interaction of IP3R1 with other regulatory proteins, including calmodulin [327]. However, it should be noted that the sites phosphorylated by PKA on IP3R1 are not conserved between the IP3R isoforms, suggesting sub-type specific differences in post-translational regulation. Having said that, there is some evidence that IP3R2 is also phosphorylated by PKA [328]. Overall, these examples highlight the multitude of ‘layers’ that are involved in IP3R regulation and the complexity of IP3-mediated calcium release.

#### Inositol 1,3,4-trisphosphate

In contrast with IP3, it is less clear if inositol 1,3,4-trisphosphate (Ins(1,3,4)P3) plays a role in calcium homeostasis, with some studies failing to show a role [329], while other studies point to a role in pressure-induced Ca(2+) waves and cerebral arterial tone in rat cerebral arteries [330] and in G-protein-coupled receptor (GPR)-54 mediated modulation of baseline calcium oscillations in neurons [331]. On the other hand, calcium itself was also shown to stimulate Ins(1,3,4)P3 production in human platelets [332]. Additionally, Ins(1,3,4)P3 production is stimulated by lithium in HL-60 leukaemia cells [333] and dog-thyroid primary cultured cells [334], by thyroid stimulating hormone (TSH) in human thyroid cells [335] and by nerve growth factor (NGF) in PC12 cells [336]. However, little is known about the functional relevance of these findings. Lastly, a recent metabolomic study has identified Ins(1,3,4)P3 as a potential serum biomarker for predicting and diagnosing coronary slow flow [337], suggesting a role in cardiovascular function.

#### Inositol 1,3,4,5-tetrakisphosphate (IP4)

To deactivate the IP3 receptors and reduce intracellular calcium store depletion, IP3 can be phosphorylated to form IP4 by IP3 3-kinases (ITPKA, ITPKB, ITPKC, IPMK/IPK2) [338]. However, in addition to being a product of targeted IP3 depletion, two distinct roles have been ascribed to IP4 itself: (i) PIP3 antagonism (and in some instances agonism) to regulate PI3K signalling and (ii) the dampening of store-operated calcium entry (SOCE) to restrict calcium mobilization. Due to the similarity of IP4 to the headgroup of PIP3, IP4 can bind to certain PIP3 effector proteins containing PH domains, thereby either promoting or inhibiting PIP3-binding to the respective effector proteins [339]. For example, while on the one hand, IP4 promotes PIP3 binding and activation of intracellular tyrosine kinase (ITK) after T cell receptor engagement [340], IP4 competition with PIP3 for binding to the PH domain of Akt limits Akt membrane recruitment and activation [341,342]. Similarly, IP4 has been shown to limit cytokine-induced Akt/mTORC1 activation in haematopoietic stem cells, ensuring stem cell quiescence and function [343], to inhibit Akt activation in permeabilized platelets, suppressing platelet aggregation [344], and to modulate Akt signalling in the context of myelopoiesis [345].

Secondly, IP4 and the kinases ITPKA/ITPKB have been implicated in SOCE activation, a ubiquitous cellular pathway for calcium influx across the plasma membrane that is dependent on a widely distributed family of ion channels, including the Ca^2+^ release-activated Ca^2+^ (CRAC) channel, and is activated in response to ER Ca^2+^ depletion (orchestrated by IP3, as discussed above) [346]. This highlights a tight and inter-connected regulation of ER and cytosolic calcium levels by both IP3 and IP4, aimed at maintaining cellular calcium homeostasis. IP4-mediated SOCE activation has been implicated in actin remodelling, contributing to tumour cell migration in various cancer models [347], in B lymphocyte selection and activation [348], as well as the hyperpolarization of erythroleukaemia cells [349]. Supporting these findings, ITPKA has a F-actin-binding domain [350] and promotes structural remodelling of dendritic spines by affecting actin dynamics [351]. Importantly, this effect is unrelated to the kinase activity of ITPKA (i.e. not directly related to IP4 production), but to the ITPKA-mediated targeting of Rac1 to the actin cytoskeleton, essential for synaptic plasticity and neural activation [351].

In addition to these two main roles for IP4, a recent study has shown that IP4 competes with NADPH for binding to NADPH oxidase 4 (NOX4), which leads to NOX4 inhibition. In this context, the authors further identified ITPKB as a central driver of cisplatin resistance in human cancers and showed that IP4 reduced cisplatin-induced oxidative stress through NOX4 inhibition [352]. These data are supported by previous studies implicating ITPKB and/or IP4 in cell survival of B cells through IP4-binding to the IP4 receptor Rasa3 [353,354], and in ERK signalling within T cells to promote cell survival [355]. However, having said that, many studies (including some mentioned above) have assessed the impact of IP3 3-kinase deletion/overexpression, which would not only lead to changes in IP4 content but also IP3 content, therefore not being able to distinguish the roles of IP3 and IP4, and/or the kinase-independent functions of the enzymes themselves. In addition, many PH domains bind PIP3 and IP4 with similar affinities [356], further pointing to the complexities surrounding the distinct role of IP4 in normal physiology.

#### Inositol 1,4,5,6-tetrakisphosphate

While inositol 1,4,5,6-tetrakisphosphate (Ins(1,4,5,6)P4) was identified almost 40 years ago [357], surprisingly little is known about this IP metabolite. In contrast with IP4, Ins(1,4,5,6)P4 does not regulate calcium homeostasis [358,359], but instead has been suggested to antagonize epidermal growth factor (EGF)-induced inhibition of chloride (Cl-) secretion in intestinal epithelial cells [360]. In this context, salmonella in intestinal epithelial cells secretes a protein called SopB, which has sequence homology to mammalian inositol polyphosphate 4-phosphatases and produces Ins(1,4,5,6)P4 [360,361], increasing chloride channel activity and cellular chloride secretion [361,362], potentially mediating the diarrhoea caused by Salmonella infection.

In addition, Ins(1,4,5,6)P4 has been suggested to act as ‘intermolecular glue’ between histone deacetylase 3 (HDAC3) and the SMRT co-repressor (also known as NCOR2) [363], pointing to an underappreciated role for Ins(1,4,5,6)P4 in transcriptional regulation. Given that HDACs are emerging cancer drug targets, this may further suggest a role for Ins(1,4,5,6)P4 in cancer pathogenesis. Indeed, Ins(1,4,5,6)P4 has been shown to inhibit membrane targeting of the PH domain of Akt and to inhibit growth of small cell lung cancer (SCLC) cells [364]. In addition, Ins(1,4,5,6)P4 binds to the PH domain to 130 kDa protein (p130) [365,366], RAC-protein kinase and diacylglycerol kinase [366], and is induced by angiotensin-II and cyclic AMP in adrenal glomerulosa cells [367], by endothelin-1 in rat fibroblasts [368] and through stimulation of the T-cell receptor in human T-lymphocytes [369], with future research required to understand if Ins(1,4,5,6)P4 has indeed a role in cancer and immune cell biology.

#### Inositol pentakisphosphate (IP5)

The importance of IP5 is highlighted by the fact that inositol polyphosphate multikinase (IPMK) knockout mice, that lose the ability to synthesize IP5, show developmental defects and die at embryonic day 9.5 [370]. This is likely related to IP5’s involvement in apoptosis and angiogenesis. Specifically, IP5 has been suggested to reduce VEGF levels and suppress vascularization (i.e. angiogenesis) through exacerbation of prolyl hydroxylation of HIF-1α, which enhances the interaction of HIF-1α with pVHL (von Hippel-Lindau protein), leading to increased HIF-1α degradation [371,372]. In addition, IP5 was shown to antagonize the PI3K pathway through inhibition of Akt, thereby inducing apoptosis across various cancer models, and additionally enhancing the proapoptotic effect of cisplatin and etoposide [373], pointing to IP5 as a specific antiangiogenic and antitumor factor [372]. Confirming a potential role in cancer, IP5 has been shown to activate HDAC1 and HDAC3, thereby likely contributing to epigenetic regulation of cancer progression [374,375] and to act as a key second messenger in cancer-associated Wnt3a signalling, including the inhibition of glycogen synthase kinase-3beta (GSK3β) and subsequent accumulation of beta-catenin [376]. Lastly, some evidence suggests that IP5 might play a role during HIV-1 infection, with HIV-1 recruiting IP5 into virions, thereby increasing HIV-1 capsid stability [377], overall promoting HIV-1 assembly and maturation [378].

#### Inositol hexaphosphate (IP6 or phytic acid)

IP6, also known as phytic acid, is abundant in cereals, legumes and nuts, and has been reported to exert beneficial effects on glucose homeostasis, inflammation and cancer prevention. In the context of glucose regulation, IP6 has been shown to lower blood glucose, HbA1c, systemic triglyceride levels and body weight in murine models of metabolic disease [379–382]. In human studies, IP6-rich diets (or IP6 supplementation) reduce postprandial glucose levels in healthy individuals, suggesting a potent role in glycaemic control [383]. Mechanistically, IP6 contributes to this effect by attenuating intestinal starch digestion through the inhibition of key polysaccharide-digesting enzymes, including α-glucosidase [384] and α-amylase [385–387]. In addition, IP6 has been reported to reduce intestinal carbohydrate absorption, further supporting a role in lowering blood glucose [379,388,389]. Similarly, combined supplementation with MI and IP6 has demonstrated synergistic glucose-lowering effects in diabetic rodent models [388–391]. These findings highlight the potential use of IP6 as a dietary strategy for the management of blood glucose control in individuals with diabetes.

Moreover, IP6 has the ability to chelate various cations, including iron [392], having the ability to reduce iron bioavailability. Indeed, dietary IP6 supplementation has been shown to inhibit protein glycation in patients with type 2 diabetes by reducing the formation of ferrous iron-catalyzed advanced glycation end products (AGEs) [382]. AGEs have been associated with various diabetes-related complications, with binding of AGEs to specific AGE receptors (RAGEs) leading to intestinal inflammation and gut permeability [393], renal and systemic inflammation [394], vascular calcification [395], arterial injury and atherosclerosis [396], among others, with AGEs contributing to increased arterial stiffness through cross-linking of collagen [397]. These data suggest that dietary IP6 may not only reduce blood glucose but also improve various diabetes complications.

Given its role as a cation chelator, IP6 supplementation has been further associated with reduced formation of various pathological calcifications, including renal [398] and dental [399] calculi, bone mass loss and probability of major osteoporotic fractures [400], as well as cardiovascular calcification [401], highlighting that dietary IP6 intake is likely to be beneficial across various clinical manifestations beyond diabetes. Supporting this idea, IP6 also shows beneficial effects in cancer [402,403], through promotion of apoptosis and inhibition of cell proliferation, survival and angiogenesis, which was attributed to suppression of the Akt/mTOR and PI3K/Akt signalling pathways [404–407].

Interestingly, IP6 supplementation has also shown promise in Parkinson’s [408,409] and Alzheimer’s disease [410]. Supporting a therapeutic opportunity for neurological disorders, IP6 can cross the blood–brain barrier, and dietary IP6 intake in rats was shown to reduce iron levels in the brain [411], confirming a role for IP6 as an ion chelator. Further studies highlight that IP6 improves cognitive function in elderly individuals [412] and that it could prevent neurological damage following cerebral ischaemia reperfusion injury, which was attributed to activation of the stress-inducible protein and antioxidant sestrin2 [413]. Due to its antioxidant and iron chelating abilities [414–416], it is not surprising that IP6 has been recently investigated in the context of ferroptosis inhibition and as a therapeutic option for ferroptosis-related diseases. IP6-containing eye drops could indeed improve oxidative stress induced-corneal epithelial injury through ferroptosis inhibition [417].

In the previous paragraphs, we touched on IP6’s ability to inhibit inflammation, with a multitude of studies highlighting a strong anti-inflammatory potential for IP6. For example, IP6 was shown to reduce pulmonary inflammation [418], neuronal inflammation after cerebral ischaemia reperfusion injury [413] and in Parkinson’s disease [408], as well as systemic proinflammatory cytokine levels [403]. In addition, IP6 enhances the expression of tumour necrosis factor receptor 1 (TNFRI) and suppresses TNF-α transcription in human colon cancer [419], while further reducing pro-inflammatory cytokines in murine macrophages [420], which may be attributed to a suppression of LPS-induced phosphorylation of IκBα, NF-κB p65, Akt and p38 both *in vitro* and *in vivo* [421], underscoring the broad anti-inflammatory potential of IP6. Given that inflammation is a major contributor to diabetes and cancer, among many other pathological states, this strongly suggests that IP6 is likely to be beneficial across a wide range of tissues and diseases.

#### Inositol pyrophosphates (IP7 and IP8)

Inositol pyrophosphates have recently emerged as important metabolites in various aspects of energy metabolism and cell signalling, with IP7 (synthesized by IP6K) and IP8 (synthesized by PPIP5K) being the primary two isoforms found in eukaryotes. In mammals, the only IP8 isomer identified to date is 1,5-IP8, while IP7 exists either as 5-IP7 or 1-IP7 (from hereon, both will be referred to as IP7). The physiological function of IP7 and IP8 is mainly related to two biochemical properties of these metabolites: (i) they act as phosphodonors contributing to protein pyrophosphorylation [422] and (ii) their pyrophosphate moiety binds to PH or C2 domains on proteins, leading to the displacement of phosphoinositides, as has been shown for the PH domain of Akt [423] and the C2 domain of synaptotagmin 7 (Syt7) [424], thereby influencing protein localization to membranes.

Syt7 is a calcium sensor important for insulin granule fusion in pancreatic beta cells, with vagal stimulation and activation of muscarinic acetylcholine receptors leading to increased IP7 generation via IP6K1 and displacement of PI(4,5)P2 on Syt7 [425]. In addition, IP7 binds calcium with high affinity, thereby freeing Syt7 to stimulate insulin granule exocytosis, with this effect diminished following beta cell-specific deletion of IP6K1 and exacerbated through expression of a hyperactive IP6K1 mutant [425]. These recent findings have been supported by previous studies showing that beta cells show higher IP6K1 expression and maintain higher IP7 levels than many other tissues [426], that glucose transiently increases beta cell IP7 content through IP6K1 and enhances insulin release [427], that deletion of IP6K1 suppresses insulin exocytosis [426] and circulating insulin levels [428], while overexpression increases insulin secretion [426]. In addition, diabetic mouse islets show reduced IP7 content and dysfunctional insulin release [427], overall supporting a distinct function for IP7 in pancreatic insulin secretion and systemic glycaemic control.

Interestingly, while IP7 on the one hand increases insulin exocytosis, on the other hand, it was shown to suppress insulin signalling in peripheral tissues, seemingly a contradictive scenario. Specifically, IP7 inhibits Akt through competitive binding with PIP3, thereby preventing PDK1-mediated phosphorylation and downstream insulin signalling [423]. Mice with IP6K1 deletion [423,429,430] or pharmacological IP6K inhibition [431–433] show improved insulin sensitivity and are resistant to diet-induced obesity, which has been attributed to AMPK activation and thermogenic energy expenditure in adipose tissue [429–431], and potentially to enhanced adipose tissue lipolysis [434]. Overall, these studies highlight that inhibition of IP6K1 in peripheral tissues may constitute a therapeutic approach to treating both obesity and diabetes.

Furthermore, biological and pharmacological inhibition of IP7 generation reduces cardiomyocyte apoptosis and myocardial ischaemia–reperfusion injury [432,435], likely through activation of the PI3K/Akt/BAD pathway and enhanced mitochondrial biogenesis [432] as well as suppression of IP7-mediated intracellular adiponectin degradation [435]. Lastly, recent reports have suggested that IP7 promotes endocytosis and downstream degradation of Na+ /K+ -ATPase, one of the most abundant cell membrane proteins, by IP7 binding to PI3K p85α to disinhibit its interaction with the sodium-potassium pump [436].

In addition to its capacity to directly bind to proteins, IP7 can act as a phosphodonor contributing to protein pyrophosphorylation, a covalent protein modification that is unique to inositol pyrophosphates, and is thought to be resistant to many known protein phosphatases [437]. For example, IP7 promotes MYC degradation through serine pyrophosphorylation of its PEST domain, which post-translationally enhances MYC polyubiquitination [438]. Moreover, IP7 binds to focal adhesion kinase (FAK), promoting its pyrophosphorylation and dimerization, which activates downstream signalling required for focal adhesion, cell motility and angiogenesis in endothelial cells [439]. In addition, pyrophosphorylation has been observed for AP3, a clathrin-associated protein complex required for HIV-1 release [440], and the dynein intermediate chain, affecting cytoplasmic dynein-driven vesicle transport [441]. Despite these few examples, protein pyrophosphorylation remains understudied, largely related to the challenging nature of mass spectrometry-based techniques for detection of protein pyrophosphorylation.

While IP7 has been shown to compete with phosphoinositides for protein binding to PH and C2 domains (as discussed above), in 2016, Wild and colleagues identified a *bona fide* inositol pyrophosphate binding domain, the SPX domain, which enables cells to interact with a multitude of proteins to regulate phosphate uptake, transport and storage [442]. Indeed, both IP7 and IP8 were shown to bind to the SPX domain of the phosphate exporter XPR1 and exacerbate the export of intracellular phosphate, with this effect diminished following expression of XPR1 harbouring a mutated inositol pyrophosphate–binding pocket [443–445]. Interestingly, a study published around the same time suggested that the regulation of XPR1 is specific to IP8, and no other inositol pyrophosphate [446], suggesting distinct, despite overlapping, functions for IP7 and IP8. Having said that, IP8 was also shown to bind to the PH domain of Akt and inhibit Akt signalling, despite with lower affinity than IP7 [447]. Given the lower affinity, conversion of IP7 to IP8 relieves the inhibitory effects of IP7 upon the PI3K signalling pathway, favouring the association of PH domain proteins with PIP3 [447].

While various studies exist assessing the impact of PPIP5K deletion, which leads to a reduction in cellular IP8, these genetic studies further frequently increase IP7 content, therefore not allowing to fully distinguish the physiological roles of IP7 and IP8 [448,449], and few studies have shown a direct role for IP8. Furthermore, the pyrophosphatase domain of PPIP5Ks can dephosphorylate the 1-position of IP8 to regenerate IP7 [450], a feedback mechanism that stabilizes intracellular IP8 levels despite fluctuations in IP7 [451,452]. This interconversion allows IP7 and IP8 to function as biochemically linked, yet relatively independent, signalling molecules.

With this in mind, only a few studies have shown direct roles for IP8 in eukaryotes, and particularly in mammals. For example, IP8 appears to act as a sensitive indicator of cellular bioenergetic stress in HCT116 tumour cells, with its levels decreasing in response to low glucose availability and glycolytic impairments, even before measurable changes in ATP content occur [449]. Similar to a role for IP7 in insulin secretion, IP8 was shown to contribute to insulin exocytosis by binding to granuphilin, a Rab effector that tethers insulin granules to the plasma membrane via SNARE and PIP2 interactions, with this binding disrupting granuphilin function and indirectly facilitating insulin granule release [453]. Future studies are required to elucidate further physiological roles for IP8 that are distinct to other inositol pyrophosphates.

## Inositol phosphate metabolism in metabolic liver disease

MASLD is intrinsically linked with metabolic conditions such as obesity and T2D, creating a complex network of co-existing disorders. With the prevalence of MASLD and MASH reaching 70% and 82% among overweight individuals, an alarming 55% and 48% among patients with T2D, and 69% and 82%, respectively, among those with dyslipidaemia [23], it is evident that these disorders are interconnected. Sedentary lifestyle coupled with a diet high in sugars and lipids create an energy surplus in the human body [454]. This excess energy is subsequently stored in the adipose tissue, leading to obesity [454], which can trigger chronic inflammation in adipose tissue, causing changes in the secretion of adipokines and pro-inflammatory cytokines and chemokines [455]. These changes pave the way for the onset of systemic insulin resistance and T2D and can also directly contribute to the development of MASLD [456]. Insulin resistance plays a dual role in this scenario. While it drives the progression of MASLD, it is also influenced by the presence of MASLD [457]. In this way, insulin resistance and MASLD fuel each other. This is of importance, as IP metabolism has been implicated in both the primary characteristics of MASLD/MASH, including hepatic steatosis and inflammation, as well as the secondary characteristics, such as hepatic insulin sensitivity.

**Figure 5:**
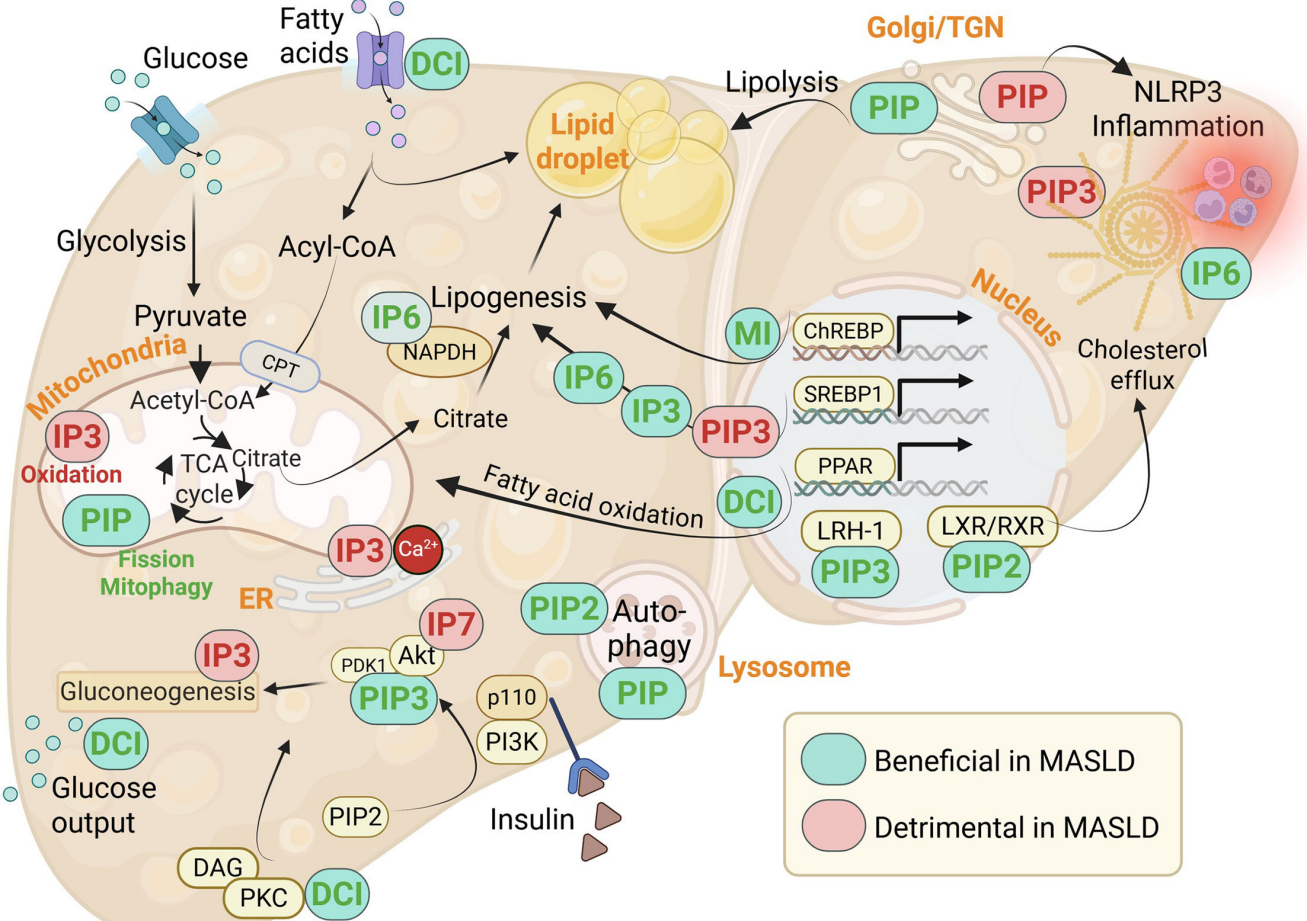
Changes in phosphoinositides and inositol phosphates (IPs) in metabolic dysfunction-associated steatotic liver disease. Summary of the major pathways that are affected by phosphoinositides and IPs in the liver in the context of metabolic dysfunction-associated steatotic liver disease (MASLD), with inositol metabolites highlighted in green suggested to have beneficial effects on the shown hepatic pathways, while inositol metabolites highlighted in red have been shown to be detrimental in the context of MASLD. ChREBP, Carbohydrate-responsive element-binding protein; CPT, Carnitine palmitoyltransferase; DAG, diacylglycerol; DCI, di-chiro-inositol; ER, endoplasmic reticulum; IP3, inositol 1,4,5-trisphosphate; IP6, phytic acid; IP7, diphosphoinositol pentakisphosphate; LRH-1, Liver receptor homolog-1; LXR, liver x receptor; MI, myo-inositol; NLRP3, NOD-, LRR- and pyrin domain-containing protein 3; PDK1, Phosphoinositide dependent protein kinase 1; PKC, protein kinase c; PIP, phosphatidylinositol 4-phosphate; PIP2, Phosphatidylinositol 4,5-bisphosphate; PIP3, phosphatidylinositol 3,4,5-trisphosphate; PPAR, Peroxisome proliferator-activated receptor; RXR, retinoid x receptor; SREBP, Sterol regulatory element-binding protein; TGN, trans golgi network

In the previous sections, we have the discussed the role of various phosphoinositides and IPs in the regulation of PI3K/Akt signalling, the generation of insulin-mimicking inositol phosphoglycans and glycosylphosphatidylinositol (GPI) anchors on proteins, as well as the direct impact of myo- and/or chiro-inositol on AMPK activity and mitochondrial function, pointing to an important role of IP metabolism in MASLD and hepatic insulin sensitivity. However, most previous studies (in the literature and discussed above) did not focus specifically on the liver, and the direct impact of the majority of phosphoinositides and IPs on MASLD progression is still elusive. The subsequent sections summarize our current understanding of changes in IP metabolism in metabolic liver disease and hepatic insulin sensitivity (summarized in Figure 5).

### Myo-inositol and D-chiro-inositol in metabolic liver disease

Decreased plasma MI levels have been reported in individuals with insulin resistance, type 2 diabetes and gestational diabetes [458–460], which is related to the finding that hyperglycaemia decreases the absorption and synthesis of MI, while further increasing its degradation and urinary excretion [6], with glucose shown to competitively inhibit inositol uptake by sodium ion-coupled transporters [461]. Early studies in rats further showed that inositol deficiency in the liver leads to accumulation of hepatic triglycerides and cholesterol [462,463] and that insulin resistance impaired the conversion of MI to DCI in skeletal muscle, adipose tissue and the liver [3].

While there are no concrete guidelines on dietary inositol intake in humans, various dietary studies in humans and rodents suggest that inositol supplementation has beneficial effects on MASLD and/or insulin sensitivity. For example, dietary MI supplementation in obese individuals with MASLD was shown to reduce fasting insulin and HOMA-IR, a measure of systemic insulin resistance and β-cell function, and to further improve the systemic lipid profile and circulating markers of liver injury (ALT/AST) [464–467], which was associated with increased expression of AMPK, Akt and PDK1 in peripheral blood mononuclear cells (PBMCs) [465]. In addition, supplementation of pinitol, a common inositol ether in plants, reduced liver fat content, plasma liver enzymes and plasma triglycerides in individuals with MASLD, while further reducing markers of oxidative stress, including urinary malondialdehyde levels [468].

Similarly, MI and/or DCI supplementation in women with polycystic ovary syndrome (PCOS), who frequently present with insulin resistance and compensatory hyperinsulinaemia, reduced circulating insulin levels, improved systemic glucose tolerance and/or insulin sensitivity, systolic/diastolic blood pressure and/or circulating lipids (triglycerides and cholesterol) [469,470] and reduced body weight [471], which was associated with improvements in the menstrual cycle across all studies. Similar beneficial metabolic effects have been observed in women with gestational diabetes [472,473] and postmenopausal women with metabolic disease [474,475]. Despite these studies not directly assessing changes in hepatic metabolism or MASLD severity, improvements in glucose tolerance and reductions in circulating insulin point to likely improvements in hepatic insulin sensitivity [476], while a reduction in fasting plasma triglyceride primarily reflects changes in secretion of very low-density lipoproteins (VLDL) from the liver [477,478]. While these studies point to inositol supplementation being beneficial in the context of metabolic disease, it is unclear if the observed improvements were directly related to increased MI content within tissues, or to increased availability of secondary inositol derivatives, including the phosphoinositides and IPs discussed here.

The findings in humans are supported by rodent data which additionally provide some mechanistic insights into the actions of inositol in the liver. For example, MI supplementation was shown to reduce liver triglyceride content and lipogenic gene expression in rats [479], due to a suppression in fructose-induced ChREBP binding to the carbohydrate response element of fatty acid synthase [480], probably leading to a reduction in hepatic *de novo* lipogenesis, one the major pathways that is dysregulated in MASLD [481]. In addition, inositol supplementation was shown to suppress the acetylation of histones H3 and H4 at the Elovl6 (elongation of very long chain fatty acids 6) promoter, contributing to reduced expression of Elovl6 [482], a fatty acid elongase and critical modulator of inflammation, oxidative stress and fibrosis in the liver [483]. The marked improvements of hepatic lipid metabolism are supported by a study showing that DCI supplementation in mice reduced hepatic fatty acid uptake and diacylglycerol deposition, while also suppressing PKCε translocation, which was associated with improved insulin sensitivity, as well as reduced gluconeogenesis and hepatic glucose output [136]. Similarly, DCI supplementation in rats reduced body weight and circulating triglyceride, cholesterol, insulin and fasting glucose levels, while also improving hepatic steatosis, which the authors attributed to activation of the Adiponectin/AMPK/PPAR transcriptional axis in the liver [484]. Taken together, these data suggest that both MI and DCI supplementation restore various metabolic pathways that have become dysfunctional in MASLD, including lipogenesis, fatty acid uptake and elongation, as well as hepatic insulin sensitivity, overall leading to marked improvements in MASLD pathogenesis and supporting the idea that inositol supplementation could be a feasible dietary approach in combination with pharmacotherapy for MASLD and/or type 2 diabetes.

### Phosphatidylinositol in metabolic liver disease

Only a small number of studies have reported changes in the abundance of hepatic PI in the context of hepatic steatosis or MASH. While a recent study suggested reduced hepatic PI in patients with MASLD [485], several other studies observed no changes in hepatic PI content [486–489]. However, despite that, dietary PI was shown to prevent the development of hepatic steatosis and inflammation in rats, which was associated with enhanced fatty acid oxidation [490]. The observed increase in fatty acid oxidation could be attributed to enhanced PI3K signalling and increased peroxisome proliferator activated receptor alpha (PPARα) transcriptional activity [491,492] and not due to an increase in hepatic PI itself. Furthermore, a genetic screen for liver defects in zebrafish showed that a lack of *de novo* PI synthesis through CDIPT (i.e. phosphatidylinositol synthase (PIS)) was associated with increased hepatic ER stress and a MASLD-like phenotype in zebrafish [493], overall pointing to hepatic PI (or PI metabolites) being protective in MASLD pathogenesis.

This is further supported by the finding that mutations in membrane-bound *O*-acyltransferase 7 (*MBOAT7*), an enzyme that diversifies the fatty acid composition of PI, have been associated with severe liver disease, including increased risk for hepatic steatosis, liver damage, hepatic fibrosis and liver cancer (reviewed in 33). Specifically, MBOAT7 is an acyltransferase that esterifies arachidonyl-CoA (C20:4) to lysophosphatidylinositol (LPI) generating the characteristic fatty acid profile of PI with predominant enrichment in stearic acid (C18:0) and arachidonic acid (C20:4) [494]. Hepatic knockdown/deletion of MBOAT7, which leads to the accumulation of its substrate LPI, exacerbates diet-induced steatosis, inflammation, cell death, fibrosis and hepatic insulin resistance in mice [495–498]. However, the authors further showed that direct administration of LPI could mimic most of the effects of MBOAT7 deletion, suggesting that the accumulation of LPI (and not the depletion of PI) is the primary driver of this phenotype [495]. The authors further showed that MBOAT7 is decreased in humans and rodents with obesity and highlighted a strong negative correlation between hepatic MBOAT7 expression with insulin resistance and obesity [495]. Similarly, plasma LPI is increased in patients with MASH and was shown to exacerbate hepatic steatosis through AMPK-mediated activation of ACC and *de novo* lipogenesis, and simultaneous suppression of mitochondrial fatty acid oxidation [499]. This was accompanied by increased hepatic expression of the LPI receptor GPR55 in MASH, with GPR55 inhibition blocking the LPI-mediated deterioration of metabolic health [499]. The LPI/GPR55 system has also been shown to increase lipogenesis in adipose tissue and to drive obesity [500]. Overall, these data highlight that while there is evidence for a role of PI in MASLD pathogenesis, it is unclear if the effects detailed above are directly linked to changes in hepatic PI or more likely related to changes in PI-derived metabolites and lipids.

### Phosphoinositides in metabolic liver disease

#### A role for PIP3 in hepatic metabolism and insulin sensitivity

While the function of many phosphoinositides has been described across diverse cell and animal models (as detailed in section 3), the role in the liver is still elusive for many of the phosphoinositides and is mostly derived or assumed from studies in other cell types and tissues. Most studies to date have focussed on describing a role for PIP3 in hepatic metabolism and insulin signalling, primarily due to its prominent role in the PI3K/Akt signalling pathway. One of the primary hepatic functions of insulin is to suppress gluconeogenesis, which is accomplished via PIP3/Akt-dependent regulation of the transcription factor Foxo1 (see section 3.3.3 for details). Specifically, the p110α catalytic subunit of PI3K is a key mediator of insulins metabolic actions in the liver, with hepatic deletion of p110α leading to reduced hepatic insulin sensitivity and an increase in gluconeogenesis [501,502], while the p110β subunit has little effect on insulin stimulation of Akt in the liver [503]. In addition, the PI3K/Akt pathway, and PIP3 more directly, have been implicated in MASLD pathogenesis. For example, activation of the PI3K/Akt pathway exacerbates hepatic steatosis due to increases in SREBP-mediated fatty acid and lipid synthesis [504–506] and drives inflammation in hepatic Kupffer cells through NLRP3 activation [507]. Mechanistically, a recent study further showed that Akt palmitoylation in MASH anchors Akt to the cell membrane, contributing to liver cancer progression. However, this was suggested to occur in a PIP3-independent manner [508]. In addition, the PI3K/Akt pathway plays an important role in the early activation of hepatic stellate cells (HSCs), contributing to cell survival, proliferation and collagen production by HSCs, with insulin showing a pro-fibrogenic effect in this context [509,510]. Overall, these data highlight that the PI3K/Akt pathway and/or PIP3 contribute to hepatic lipid deposition, inflammation and fibrosis, while on the other hand suppressing hepatic gluconeogenesis and glucose output, showing both favourable and detrimental metabolic effects in the same disease context.

While phosphoinositides primarily exist in a lipid-bound form, several studies have shown a role for phosphoinositides, and particularly PIP3, within the nucleus [511]. The liver receptor homolog-1 (LRH-1; encoded by *NR5A2*), a nuclear hormone receptor with high expression in the liver, binds PIP3 with high affinity [512] and plays an essential role in hepatic lipid storage. Hepatic deletion of LRH-1 leads to imbalanced arachidonic acid-phospholipid metabolism due to repression of *Elovl5* and *Fads2* and increased hepatic steatosis and liver injury [513], while activation of LRH-1 improves fatty liver [514]. Similarly, impaired incorporation of arachidonic acid into membranes has been previously associated with a suppression of lipoprotein metabolism and fatty liver [515]. Interestingly, patients with MASLD and MASH show reduced arachidonic acid-containing phospholipids [516] and reduced hepatic LRH-1 expression [517].

It has been stipulated that the tumour suppressor and PIP3-phosphatase PTEN, which converts PIP3 to PIP2 on the cell membrane and is a negative regulator of the PI3K/Akt pathway [518], might also remodel PIP3 bound to LRH-1 to inhibit LRH-1 activity [511]. In this respect, studies using LRH-1 activation closely phenocopy PTEN loss in the liver, with hepatic deletion of PTEN increasing fatty acid synthesis and fatty liver [519] and contributing to MASH and liver cancer progression [520]. On the other hand, hepatic PTEN knockout mice show enhanced PI3K/Akt signalling and hepatic insulin action [519], highlighting a dis-connect between hepatic steatosis and insulin action in the liver. These findings are highly relevant as PTEN is inactivated in 40-50% of human liver cancers [521], leading to cancer progression through over-activation of the PI3K/Akt pathway [522]. Future research is required to fully elucidate the role of PIP3 in the nucleus and the physiological relevance of the PIP3/LRH-1 axis in MASLD.

#### A role for PIP2 in cholesterol metabolism and metabolic liver disease

While less is known about the role of PIP2 in hepatic metabolism and MASLD pathogenesis, some evidence also suggests a role for PIP2 in the nucleus (as discussed above for PIP3). In this respect, cholesterol loading in hepatocytes was shown to increase nuclear PIP2 localization and PIP2 interaction with the LXR-RXR complex to regulate the expression of ABCA1, an important regulator of cholesterol efflux, overall maintaining cellular cholesterol homeostasis [523]. Given that cholesterol homeostasis plays an important role in the pathogenesis of MASLD, and ABCA1 expression has been shown to be significantly decreased in patients with MASH and liver fibrosis [524], this points to an important (and potentially underappreciated) role for nuclear PIP2 in cholesterol efflux and MASH pathogenesis. This is further supported by the finding that the LDL receptor-related protein 1 (LRP1), which also controls hepatic cholesterol efflux and protects against diet-induced hepatic steatosis [525], recruits PIP5KL1 and PIP5K1β to the plasma membrane for PIP2 synthesis [526]. Furthermore, knockdown of the PIP2 phosphatase TMEM55B, which is associated with increased PIP2 levels, increases lysosomal degradation of the LDL (low-density lipoprotein) receptor, thereby suppressing hepatic cholesterol uptake [527]. Addition of exogenous PIP2 mimicked the effects of TMEM55B knockdown [527], with these studies overall suggesting a direct role for PIP2 in both the promotion of cholesterol efflux and the suppression of cholesterol uptake.

#### A role for PIP in metabolic liver disease

Various MASLD-related studies suggest an important role for PIP within the Golgi. For example, hepatic glucose depletion results in lower PIP content within the Golgi, which has been shown to reduce polyubiquitylation of ATGL, thereby enhancing lipolytic fatty acid release both for intracellular use and for extracellular secretion [528]. Importantly, the authors further show that PIP within the Golgi might serve as a potent target to block MASH progression, primarily due to its impact on lipolysis [528]. However, PIP within the TGN has also been suggested to recruit NLRP3, leading to NLRP3 aggregation and downstream inflammatory signalling [529], with inhibition of NLRP3 improving MASLD pathology and fibrosis in obese diabetic mice [530]. These findings suggest that increases in hepatic and particularly Golgi-localized PIP might reduce hepatic steatosis but simultaneously exacerbate pro-inflammatory signalling.

Recently, golgi phosphoprotein 3 (GOLPH3) has additionally been identified as an important PIP effector in the Golgi, required for maintenance of Golgi architecture and vesicular trafficking [531]. While little is known about the GOLPH3/PIP axis in MASLD progression, GOLPH3 has been shown to promote renal fibrosis [532] and hepatocellular carcinoma [531], suggesting that the GOLPH3/PIP axis might have an uncharacterized role in hepatic fibrosis. In addition, PIP within the trans Golgi network plays an important role in mitochondrial division, with PIP depletion leading to a hyperfused and branched mitochondrial network [533], mitochondrial fragmentation [534] and mitophagy [535], with PIP also shown to be important for cardiolipin synthesis [526]. Given that mitochondrial dysfunction is a major characteristic of MASLD [536], future studies are required to elucidate the role of PIP within the Golgi in MASLD progression. A recent study also suggested a role for PIP in clathrin recruitment and tubule budding during autophagic lysosome formation in the liver [537], a critical lysosomal pathway that maintains cellular function and cell survival but may also promote the development of hepatic fibrosis [538]. Supporting a role for PIP in autophagy and metabolic liver disease, deletion of Pip4k2a and Pip4k2b, important in PIP2 synthesis from PIP, leads to defects in autophagy and exacerbates hepatic steatosis [32]. Future studies are warranted to dissect the impact of hepatic PIP in the various aspects of MASLD pathology, including mitochondrial dysfunction, autophagy, hepatic steatosis and inflammation.

#### A role for PI(3,4)P2 and PI(3,5)P2 in metabolic liver disease

Little is known about the role of PI(3,4)P2 and PI(3,5)P2 in the context of metabolic liver disease. PI(3,4)P2 has been shown to promote Akt2 phosphorylation [539–541] and thereby regulate glucose homeostasis [540] as well as cytoskeletal reorganization during the progression of liver cancer [539]. To our knowledge, the only evidence for a role of PI(3,5)P2 in liver disease comes from a recent study in zebrafish, showing that hepatic PI(3,5)P2 deficiency leads to abnormal lysosomal storage with accumulation of autophagy intermediates [542]. While lysosomal PI(3,5)P2 has been shown to terminate PI3K activity via specific interaction with its regulatory p85 subunit in neurons [283], to our knowledge a specific role for PI(3,5)P2 in hepatic PI3K signalling has not been described.

### Soluble inositol phosphates in metabolic liver disease

#### A role for inositol 1,4,5-trisphosphate (IP3) and IP3 receptors in metabolic liver disease

Various physiological functions within the liver (e.g. cell proliferation, gene expression, cell death) are dependent on intracellular calcium signalling, and with the IP3 receptor being the only calcium release channel within the liver [543], it is not surprising that IP3 plays an important role in hepatic physiology and pathophysiology. Within the liver, IP3R1 and IP3R2 are the predominant isoforms in hepatocytes [544] while cholangiocytes express all three isoforms [545]. Hepatic IP3R1 expression is increased in ob/ob mice with type 2 diabetes and in mice with diet-induced obesity [546], as well as in patients with MASH [547]. IP3R1 is primarily localized to mitochondria-associated ER membranes (MAMs), ER-mitochondria contact sites, that play an important role in nutrient exchange and calcium signalling [546]. Increased IP3R1 expression exacerbates calcium release from the ER and drives progression of mitochondrial dysfunction and impairments in glucose homeostasis [546], particularly increasing the capacity of glucagon to stimulate hepatic gluconeogenesis [548,549]. On the other hand, depletion of IP3R1 from MAMs improves mitochondrial oxidative capacity and glucose metabolism in obese mice [546]. Mechanistically, HCLS1-associated protein X-1 (HAX-1) interacts with IP3R1, exacerbating calcium overload and impairing mitochondrial fatty acid and glucose oxidation. In contrast, HAX-1 deletion enhances mitochondrial glucose and fatty acid oxidation, subsequently preventing hepatic steatosis and insulin resistance [550]. Similarly, IR3R1 knockout mice are resistant to the development of hepatic steatosis [547]. Overall, these data highlight that IP3R1 (and/or HAX-1) may represent a therapeutic target for MASLD and type 2 diabetes.

In contrast with IP3R1, IP3R2 expression is decreased in obese mice [551,552] and in patients with steatosis and MASH [552], with expression probably regulated through the transcriptional factor c-Jun [552]. In addition, the ITPR2 rs11048570 variant has been associated with a reduced risk of MASLD [553]. The physiological function of IP3R2 in MASLD and metabolism has not been fully elucidated given conflicting data from knockout mouse models. In this respect, while one study showed that IP3R2 knockout mice have no apparent metabolic phenotype, including no changes in glucose production, glucose tolerance or susceptibility to hepatic steatosis [551], an independent study pointed to knockout mice showing less liver steatosis and fibrosis, as well as better response to metabolic stress [554]. In addition, a recent study showed that the hepatokine orosomucoid (ORM) 2 binds to IP3R2, leading to the activation of AMPK signalling and suppression of SREBP-mediated lipogenic gene expression in the liver, with ORM2 protein therapy improving hepatic steatosis and MASH in mice [555]. These data suggest that targeting IP3R2 (and/or ORM2) may have beneficial effects in the liver. Despite differences in feeding conditions, age and interventions between these studies, the contradicting outcomes require future validation.

From the above summary on the role of IP3 in the liver, it is evident that the vast majority of studies investigated the role of IP3 receptors in metabolic liver disease, with very few studies assessing changes in actual IP3 content in the liver and/or the role of IP3 (dependent or independent of IP3R signalling) in metabolic liver disease, and future studies are required to distinguish the physiological importance of IP3 from the role of IP3 receptors.

#### A role for inositol 1,3,4,5-tetrakisphosphate (IP4) and inositol pentakisphosphate (IP5) in metabolic liver disease

In comparison with IP3, to our knowledge very few studies have focussed on IP4 in the liver and its role in metabolic liver disease. Early studies from the 1980’s and 1990’s have shown that IP4 is present in the liver, that it is synthesized from IP3 and has the ability to sequester calcium [556,557], that it prolongs the duration of the calcium transient induced by IP3 in the liver [558], and that IP4 mediates nuclear calcium signalling through the IP4 receptor localized to the outer nuclear membrane [559–561]. Similar to IP4, very little is known about the role of IP5 in the liver. Early studies have suggested that IP5 can also stimulate nuclear calcium influx [562]. In addition, it has been suggested that IP3 kinase (ITPKA) expression is increased in liver cancer and associated with poorer patient prognosis and survival [563], pointing to liver cancer potentially showing an increase in IP4 content. However, to our knowledge there are no studies investigating changes in IP4 or IP5 content in MASLD and/or the role of IP4 and IP5 in the pathogenesis of metabolic liver disease.

#### A role for inositol hexaphosphate (IP6 or phytic acid) in metabolic liver disease

Phytic acid has been shown to have protective effects in many pathological conditions, including MASLD. Dietary phytic acid supplementation alleviates hepatic steatosis, inflammation and fibrosis across various rodents models [35,36,564–567], with several studies highlighting that the suppression in hepatic lipid accumulation is related to reductions in lipogenic gene expression [564,565,568–570]. Mechanistically, IP6 supplementation has been suggested to reduce the activity of glucose-6-phosphate dehydrogenase and malic enzyme in the liver [570], with both enzymes providing NADPH for lipogenesis [571,572]. In addition, supplementation with a cerium-phytic acid nanocomplex led to reduced hepatic mTOR activity, thereby suppressing SREBP1 transcriptional activity [573], an important transcriptional regulator of lipogenic gene expression [574]. Phytic acid was further shown to ameliorate inflammation and oxidative stress in the liver [34,391,575,576] and to reduce both circulating cholesterol [35,390,567,570] and triglycerides [381,390,570], overall showing protective effects in the liver. In humans, while IP6 supplementation has been shown to be beneficial in various cancers [577], chronic kidney disease [578] and short bowel syndrome [579,580], and while animal studies show beneficial effects in the context of MASLD, to our knowledge little is known about the impact of dietary IP6 supplementation on liver function and MASLD in humans.

#### A role for inositol pyrophosphates (IP7 and IP8) in metabolic liver disease

While inositol pyrophosphates have been shown to affect a wide range of physiological processes through their ability to act as phosphodonors in protein pyrophosphorylation and through direct binding to PH or C2 domains on proteins, little is known about their role in the liver and changes in IP7/IP8 content in MASLD. While expression of IP6K3 (one of the kinases that generates IP7) has been shown to be increased in MASLD [581] and increased expression of IP6K1 and IP6K2 has additionally been associated with liver cancer [582], actual changes in IP7 or IP8 have not been investigated in these studies. Furthermore, various previous studies suggested that deletion or pharmacological inhibition of IP6K ameliorates hepatic steatosis [433], which has been associated with reduced expression of markers of fatty acid uptake and lipogenesis [583] as well as augmented Akt, mTOR and GSK3β signalling in the liver [423,429,431]. In addition, IP6K inhibition and/or deletion improves glycaemic control and reduces whole-body adiposity, potentially by boosting Akt signalling and mitochondrial oxidative capacity in both adipose tissue and liver [423,583]. Mechanistically, IP7 has been suggested to inhibit the PDK1 phosphorylation of Akt, preventing Akt activation and thereby suppressing insulin signalling [423]. However, similarly these studies focussed on the kinases that generate IP7 and did not show direct effects of modulating inositol pyrophosphates in the liver. Future research is required to gain a better understanding of the direct role of IP7 and IP8 in hepatic metabolism and MASLD pathogenesis.

### Gut microbiota dysbiosis in MASLD – Impact on inositol phosphate and phosphoinositide metabolism

Various studies in mice and patients with MASLD have described changes in the composition and function of gut microbiota, also referred to as dysbiosis. Specifically, MASLD has been associated with (i) a reduction in the diversity of gut microbiota, (ii) reduced levels of beneficial Gram-positive bacteria, mostly Firmicutes, and (iii) a simultaneous increase in pathogenic Gram-negative bacteria, primarily Proteobacteria [584]. However, it should be noted that many previous studies did not take into account cofounding factors, including presence of obesity, type 2 diabetes, as well as age, sex and/or ethnicity [585], and it is unclear if these associations are directly related to MASLD, or, for example, obesity and/or differences in dietary intake [586]. Nevertheless, the remodelling of the gut microbiome in individuals with MASLD has been shown to lead to a pro-inflammatory environment and drive gut barrier dysfunction, contributing to the release and subsequently hepatic exposure of microbiota-derived factors, including lipopolysaccharide (LPS), peptidoglycan, viral or bacterial DNA, or fungal beta-glucan, among others [586]. These components are recognized by immune cells within the liver and chronic exposure can lead to inflammation and fibrosis.

While studies on the direct impact of microbiota-derived factors on MASLD progression are scarce, LPS has been shown to bind to cell surface proteins in macrophages, including CD14 [587] and the TLR4/MD2 receptor complex [588], leading to LPS-induced palmitoylation of PI4KIIβ and increased generation of PIP2 [589,590], which is likely to contribute to hepatic steatosis [32], tumour metastasis [31] and the maintenance of cellular cholesterol homeostasis [523]. Additionally, PIP2 is a substrate for the synthesis of IP3 and PIP3, with these enzymatic steps required for the internalization of TLR4/MD2 and initiation of downstream signalling [591,592]. While LPS also activates TLR4/CD14 signalling in Kupffer cells, exacerbating inflammation, oxidative stress and apoptosis [593], there is to our knowledge no direct evidence that LPS affects IP metabolism in this setting. In cholangiocytes, LPS stimulates TLR4 and activates NF-κB, leading to decreased expression of ITPR3 expression, which impairs calcium release and may contribute to cholestasis [594]. While similar to LPS peptidoglycan is recognized by TLR4 and mediates inflammatory responses [595], the direct impact of peptidoglycan on inositol metabolism in the liver is currently unknown. Similarly, while unmethylated CpG motifs in bacterial DNA are recognized by tolllike receptor 9 (TLR9) in Kupffer cells and hepatic stellate cells, contributing to IL1β [596] and collagen [597] release, respectively, there is no direct evidence that inositol metabolism is involved in these pathways.

### Inositiol phosphate metabolism in metabolic and alcohol-associated liver disease (Met-ALD)

Accompanying pure MASLD, a new category termed MetALD describes individuals with MASLD who consume greater amounts of alcohol (e.g. > 140 g/week and 210 g/week for females and males, respectively) [598]. Given that this new nomenclature has only recently come into effect, to our knowledge no studies have directly assessed changes in IP metabolism in the setting of MetALD as yet. Similarly, literature on IP and phosphoinositide metabolism in alcoholic liver disease (i.e. the old terminology) is scarce. Ethanol intake contributes to hepatic steatosis through several inter-related mechanisms, including suppression of fatty acid oxidation with simultaneous exacerbation of lipogenesis, which has been associated with defective PI3K/Akt/mTOR signalling [599]. Specifically, ethanol has been suggested to increase the association of PTEN with the PI3K p85alpha subunit leading to the inhibition of downstream insulin signalling [600]. Interestingly, MI supplementation in rats has been shown to completely abolish the increase in liver lipids induced by alcohol [601], suggesting potential benefits of MI in both MASLD and MetALD. Further studies have indicated that ethanol exposure activates polyphosphoinositide-specific phospholipase C in the liver, increasing formation of IP3 [602] and inducing release of calcium from intracellular stores [602–604], while simultaneously reducing sensitivity of the IP3 receptor [605]. Lastly, one study suggested that ethanol also increases hepatic PIP synthesis [606], therefore probably contributing to PIP-mediated changes in vesicular trafficking, membrane dynamics and sphingolipid metabolism, as discussed in the previous sections. Future studies are required to increase our understanding on the impact of hepatic ethanol metabolism and the presence of MetALD on IP and phosphoinositide metabolism in the liver (and beyond).

## Conclusion

In conclusion, free inositol, phosphoinositides and soluble IPs play pivotal roles in the regulation of cellular signalling, membrane dynamics and metabolic homeostasis, functions that are particularly crucial in the liver. Their involvement in hepatic glucose and lipid metabolism, insulin signalling and mitochondrial function underscores their central role in maintaining (or disrupting) liver physiology. Emerging studies also implicate dysregulation of these signalling molecules in the pathogenesis of MASLD, where altered IP metabolism contributes to hepatic steatosis, inflammation and insulin resistance. For example, while MI and IP6 supplementation has proven beneficial in MASLD and diabetes, we have a very limited understanding of the role of IP4 and IP5, as well as the function of inositol pyrophosphates in the liver. Continued investigation into the complex networks governed by these molecules will not only deepen our understanding of liver biology but is also likely to uncover novel therapeutic targets for MASLD and related metabolic disorders.

## Abbreviations

AGEs: Advanced glycation end products
AMPK: AMP-activated protein kinase
ARF6: ADP-ribosylation factor 6
Ago2: Argonaute 2
CDIPT: CDP-diacylglycerol–inositol 3-phosphatidyltransferase
CDIPT: CDP-diacylglycerol–inositol 3-phosphatidyltransferase
DAG: Diacylglycerol
DCI: D-chiro-inositol
DIPP: Diphosphoinositol polyphosphate phosphohydrolase
DRP1: Dynamin-related protein 1
DRP1: Dynamin-related protein 1
ER: Endoplasmic reticulum
FAK: Focal adhesion kinase
FAPP: Four-phosphate-adaptor protein
GEF: Guanine nucleotide exchange factors
GLUT: Glucose transporter
GOLPH3: Golgi phosphoprotein 3
G6P: Glucose-6-phosphate
GPCR: G protein-coupled receptor
GPI: Glycosylphosphatidylinositol
GPI: Glycosylphosphatidylinositol
GRP1: General receptor for 3-phosphoinositides 1
GSK3: Glycogen synthase kinase 3
GSK3: Glycogen synthase kinase 3
HAX-1: HCLS1-associated protein X-1
HDL: High-density lipoproteins
HMIT: H+-myo-inositol transporter
HSC: Hepatic stellate cells
HUVEC: Human vascular endothelial cells
IKK: Inhibitory-κB kinase
IMPA 1/2: Inositol monophosphatase 1/2
INPP1: Inositol polyphosphate-1-phosphatase
INPP5: Inositol polyphosphate-5-phosphatase
INPP5: Inositol polyphosphate-5-phosphatase
IP: Inositol phosphates
IP3: Inositol 1,4,5-trisphosphate
IP6: Inositol hexakisphosphate
IP7: Diphosphoinositol pentakisphosphate
IP7: Diphosphoinositol pentakisphosphate
IP8: Bis-diphosphoinositol tetrakisphosphate
IPG: Insulin-mimicking inositol phosphoglycan
IPG: Insulin-mimicking inositol phosphoglycan
IP5-2K: Inositol 1,3,4,5,6-pentakisphosphate 2-kinase
IPMK: Inositol polyphosphate multikinase
IPP: Inositol pyrophosphates
IPPK1: 1,3,4-trisphosphate 5-kinase 1
IPPK1: 1,3,4-trisphosphate 5-kinase 1
IP3R 1/2/3: IP3 receptor 1/2/3
ISYNA1: Inositol-3-phosphate synthase
ITK: Intracellular tyrosine kinase
ITPK1: Inositol 1,3,4-trisphosphate 5/6-kinase
Ins(3)P: Inositol-3-phosphate
Ins(4)P: Inositol 4-monophosphate
Ins(1,3,4,5)P4: Inositol 1,3,4,5-tetrakisphosphate
Ins(1,3,4,5)P4: Inositol 1,3,4,5-tetrakisphosphate
Ins(1,3,4,5,6)P5: Inositol 1,3,4,5,6-pentakisphosphate
KRAP: Kras-induced actin-interacting protein
LPI: Lysophosphatidylinositol
LRP1: Liver receptor homolog 1
LTP: Lipid transport proteins
MAPK: Mitogen-activated protein kinase
MASH: Metabolic dysfunction-associated steatohepatitis
MASLD: Metabolic dysfunction-associated steatotic liver disease
MBOAT7: Membrane-bound O-acyltransferase 7
MI: Myo-inositol
MI: Myo-inositol
NOX4: NADPH oxidase 4
NOX4: NADPH oxidase 4
ORP 5/8: Oxysterol-binding protein-related protein 5/8
OSBP: Oxysterol-binding protein
OSBP: Oxysterol-binding protein
PBMCs: Peripheral blood mononuclear cells
PBMCs: Peripheral blood mononuclear cells
PBMCs: Peripheral blood mononuclear cells
PCOS: Polycystic ovary syndrome
PCOS: Polycystic ovary syndrome
PDH: Pyruvate dehydrogenase
PH: Pleckstrin homology
PH: Pleckstrin homology
PI4K: PI 4-kinase
PI4K: PI 4-kinase
PI(3)P: Phosphatidylinositol-3-phosphate
PI(3)P: Phosphatidylinositol-3-phosphate
PI(4)P: Phosphatidylinositol-4-phosphate
PI(4)P: Phosphatidylinositol-4-phosphate
PIP5K1: PI4P 5-kinase
PI(4,5)P2 (PIP2): Phosphatidylinositol 4,5-bisphosphate
PI(4,5)P2 (PIP2): Phosphatidylinositol 4,5-bisphosphate
PI(4,5)P2 (PIP2): Phosphatidylinositol 4,5-bisphosphate
PI(3,4,5)P3 (PIP3): Phosphatidylinositol (3,4,5)-trisphosphate
PIS: Phosphatidylinositol synthase
PIS: Phosphatidylinositol synthase
PIS: Phosphatidylinositol synthase
PITP: Phosphatidylinositol transfer proteins
PITP: Phosphatidylinositol transfer proteins
PKA: Protein kinase A
PKC: Protein kinase C
PKG: Cyclic GMP-dependent protein kinase
PLC: Phospholipase C
PPAR: Peroxisome proliferator activated receptor
PP2C: Protein phosphatase 2C
PPIP5K: Diphosphoinositol pentakisphosphate kinase
PX: Phox homology
ROS: Reactive oxygen species
SMIT 1/2: Sodium-myo-inositol transporter
SNP: Single nucleotide polymorphism
SNX5: Sorting nexin 5
SOCE: Store-operated calcium entry
STX: Syntaxin
SYT7: Synaptotagmin 7
T2D: Type 2 diabetes
TGN: Trans-Golgi network
TLR9: Toll-like receptor 9
TNFR1: Tumor necrosis factor receptor 1
TPC: Two-pore channel
TRPML: Mucolipin transient receptor potential
TSC: Tuberous sclerosis complex
3′ UTR: 3′ untranslated region
VAMP7: Vesicle-associated membrane protein 7
VCP: Valosin containing protein
VLDL: Very low-density lipoproteins
WIPI: WD-repeat protein interacting with phosphoinositides
mTORC2: mTOR Complex 2
